# Supplementary material for: Use of polyadenosine tail mimetics to enhance mRNA expression from genes associated with haploinsufficiency disorders
Source: Mol Ther Nucleic Acids. 2025 Jan 13;36(1):102453. doi: 10.1016/j.omtn.2025.102453 (PMC11834087; doi:10.1016/j.omtn.2025.102453)
Supplement: Document S1. Figures S1–S6 and Tables S1–S4 [file mmc1.pdf]

OMTN, Volume *36*

Supplemental information

Use of polyadenosine tail mimetics to enhance mRNA expression from genes associated with haploinsufficiency disorders

Bahareh Torkzaban, Yining Zhu, Christian Lopez, Jonathan M. Alexander, Jingyao Ma, Yongzhi Sun, Katharine R. Maschhoff, Wenqian Hu, Michele H. Jacob, Dingchang Lin, Hai-Quan Mao, Sophie Martin, and Jeff Coller

Supplemental material

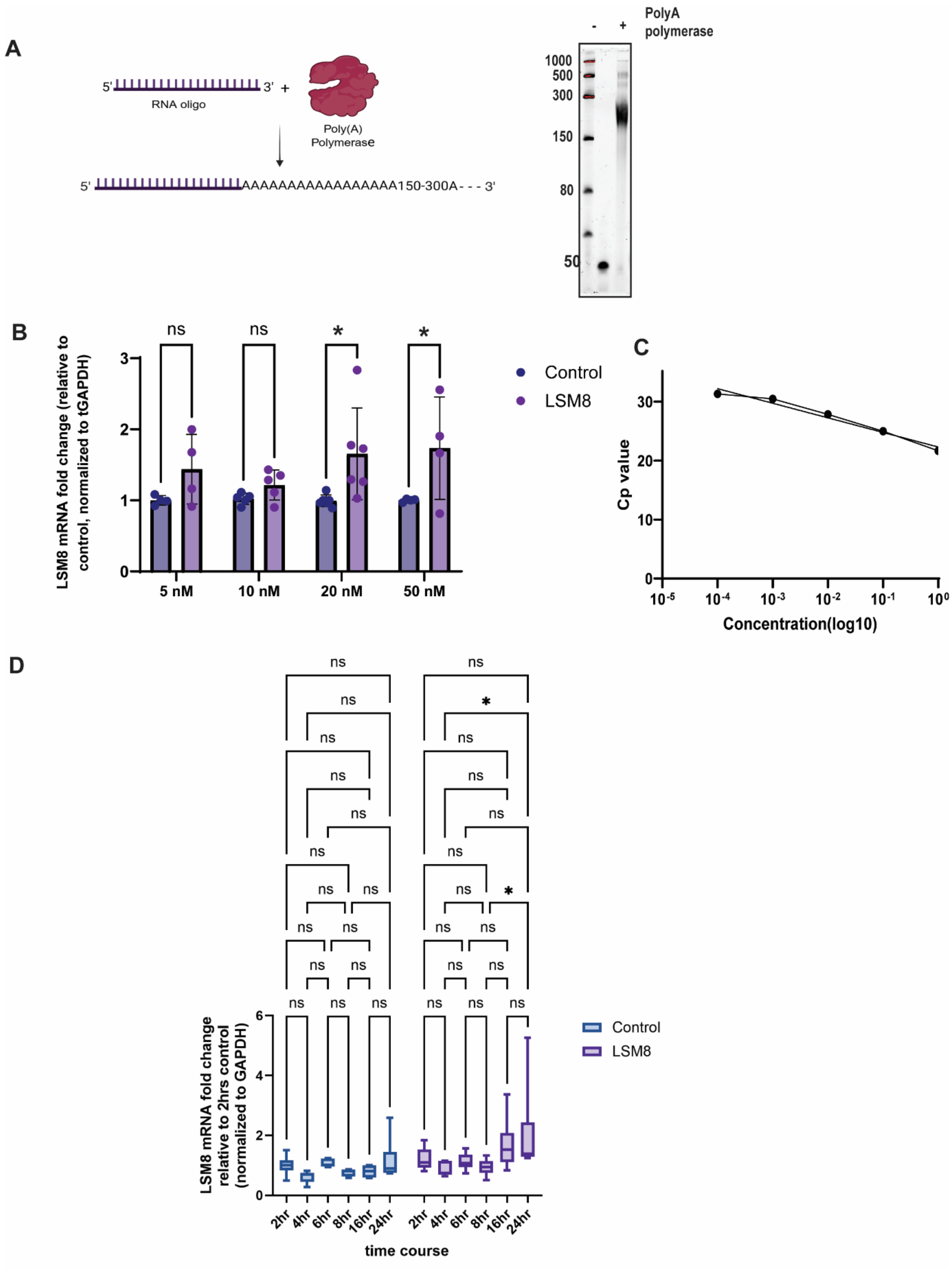

Figure S1: (A) Synthesis and quality control of boosters generated using an enzymatic approach that leads to a poly(A) tail of about 150-200 nucleotides; the poly(A) tail was added to the 3'UTR of oligonucleotides with E.Coli poly(A) polymerase, and the polyadenylated oligonucleotides analyzed by polyacrylamide gel electrophoresis. **(B)** HEK293 cells exposed to different doses of LSM8 specific and non-specific booster. 16 hours after transfection, mRNA analysis using RT-qPCR indicates the most effective dose to investigate booster activity for LSM8. **(C)** the graph shows LSM8 primer pair efficiency. **(D)** The box plot indicates no significant time dependent alteration in the level of LSM8 comparing all the control treated time points normalized to 2hr control, while there is significant alteration in the level of LSM8 when normalized to 2hr control between 4 and 24 hrs and 8 and 24 hrs booster treatment. two -way ANOVA,* *p*-value = 0.05.

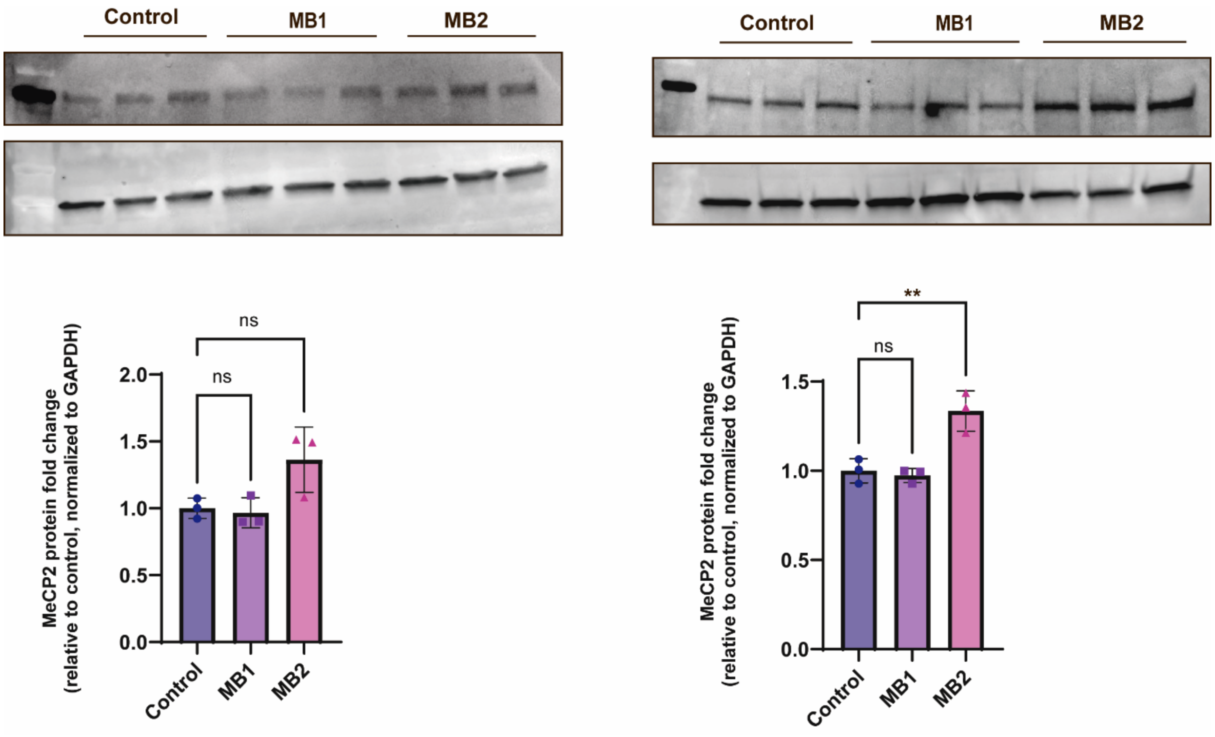

Figure S2: Western blotting technical replicates for MeCP2 from liver lysates of 6 weeks-old mice injected with 25ug of LNP-encapsulated boosters (version V2, MB1 and MB2, or non-specific Control) by tail vein injection. The tissues were collected 48 hours after injection. Ordinary one-way ANOVA, * *p*-value = 0.05,** *p*-value = 0.005.

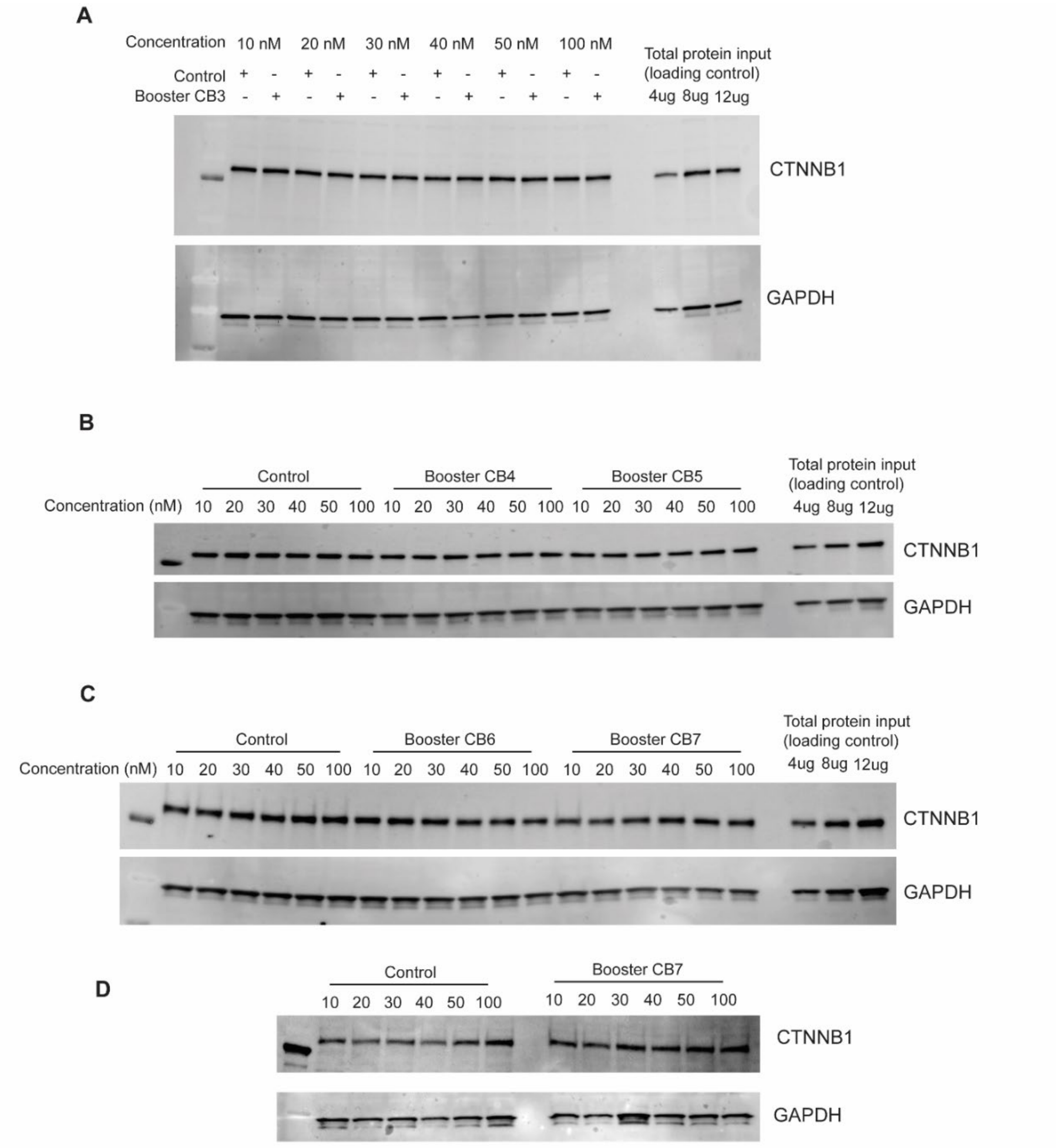

Figure S3: The western blot shows CTNNB1 protein level in HEK293 cells. HEK-STF cells treated with different doses of CTNNB1 boosters V.1.0; CB3 **(A)**, CB4 and CB5 **(B)**, CB6 and CB7 **(C)**. Cells were harvested 48 hours after transfection and the protein lysate analyzed by western blotting. Quantitation's for these blots are presented in Figure 4B. **(D)** Additional Western blot representative of CTNNB1 Booster dose response experiment.

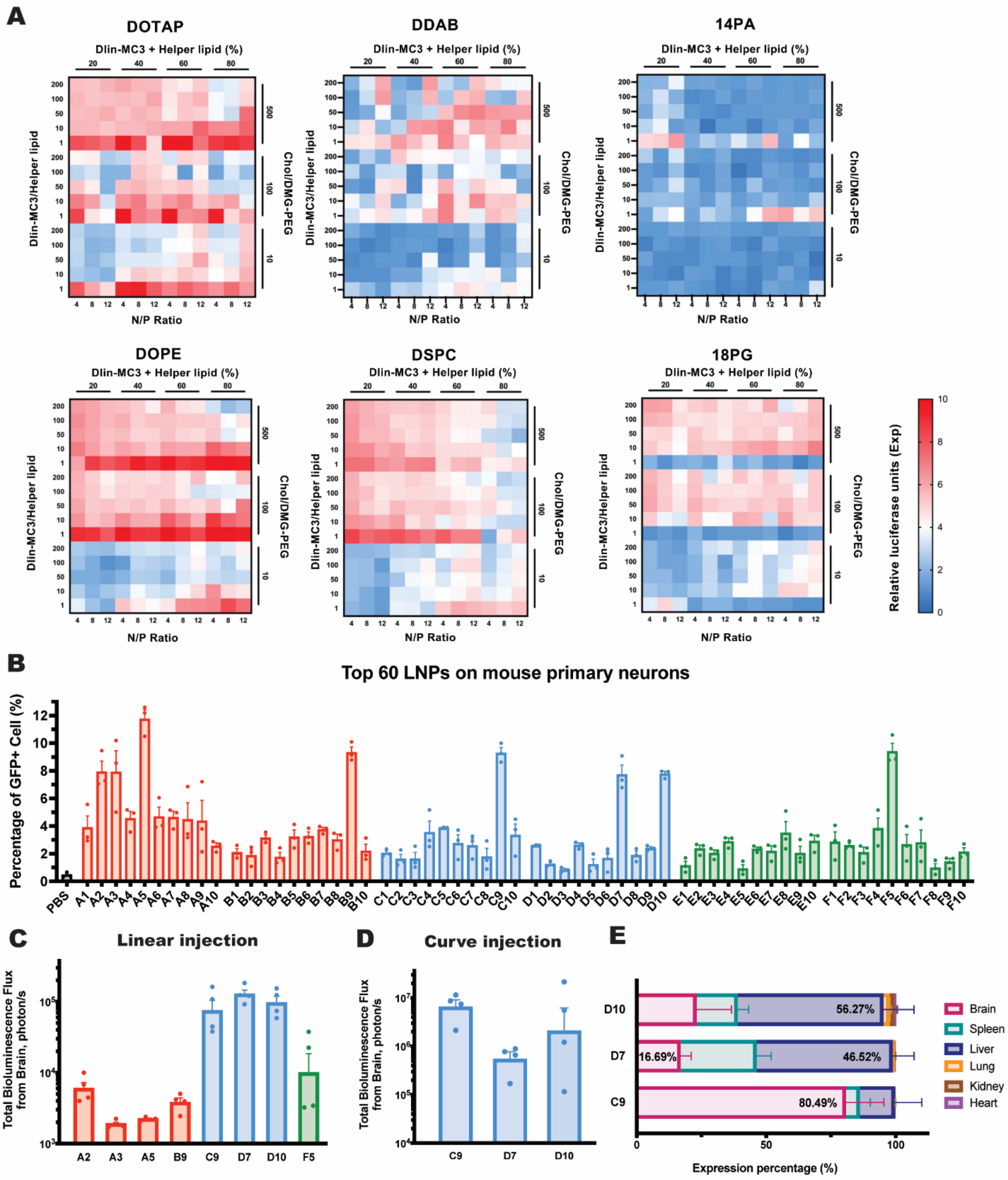

Figure S4: (A) Transfection efficiency of pDNA LNPs on Neuro-2a via high-throughput screening platform after 72h incubation. (n= 2) The efficiency of transgene expression of luciferase as a reporter. (pDNA 1μg/well) **(B)** Transfection efficiency of mRNA LNPs on

primary neurons was evaluated via flow cytometry after 24h incubation. (n= 3) The efficiency of transgene expression of GFP as a reporter (mRNA 0.5 μg/well). **(C)** Transfection efficiency of selected mRNA LNPs in the brain via linear injection was evaluated via IVIS after 24h incubation. (n= 4) The efficiency of transgene expression of luciferase as a reporter. (mRNA 5 μg per mouse) **(D-E)** Transfection efficiency of selected mRNA LNPs in the brain via circular injection was evaluated via IVIS after 24h incubation. (n= 4) The efficiency of transgene expression of luciferase as a reporter. (mRNA 5 μg per mouse).

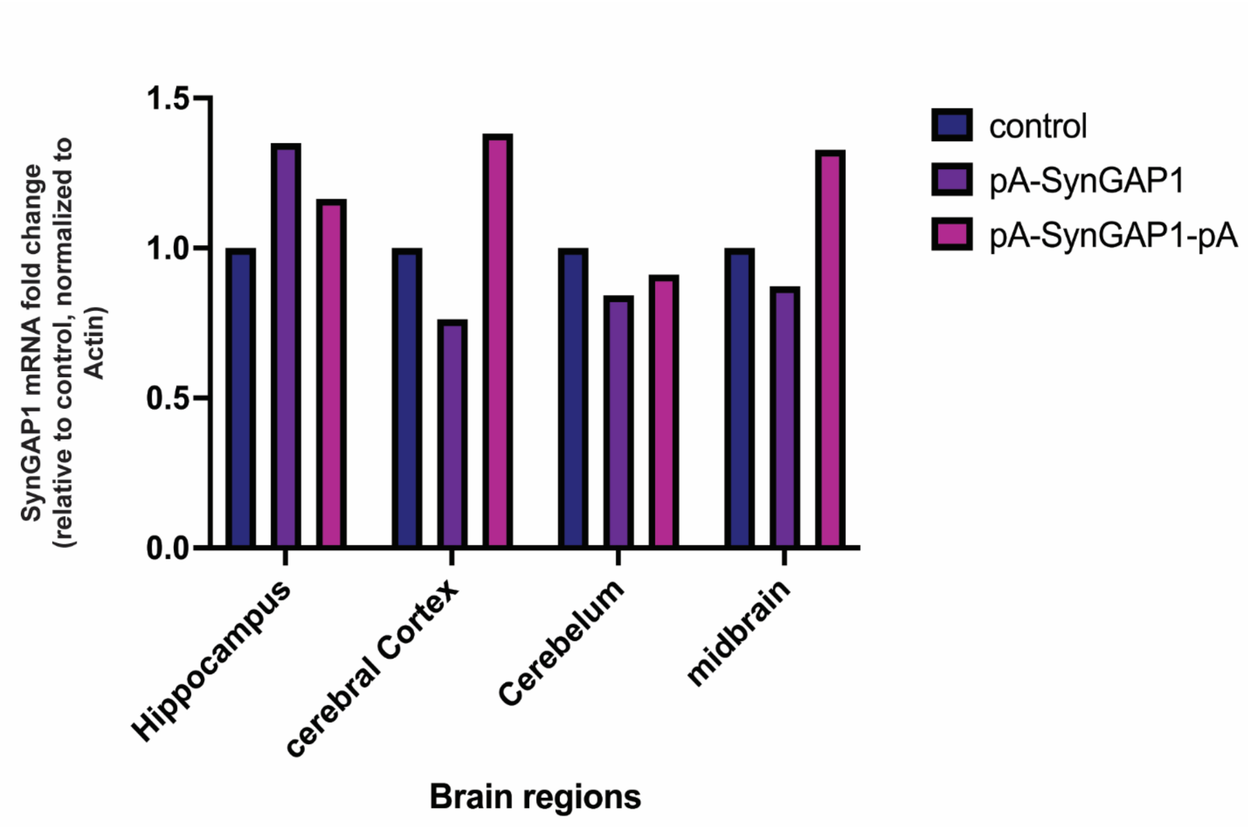

Figure S5: mRNA levels of SynGAP1 in the hippocampus of mice injected with 25 ug of LNP-packaged SynGAP1 boosters V.2.0 (SB1 and SB2) or a non-specific scrambled control, measured by RT-qPCR analysis.

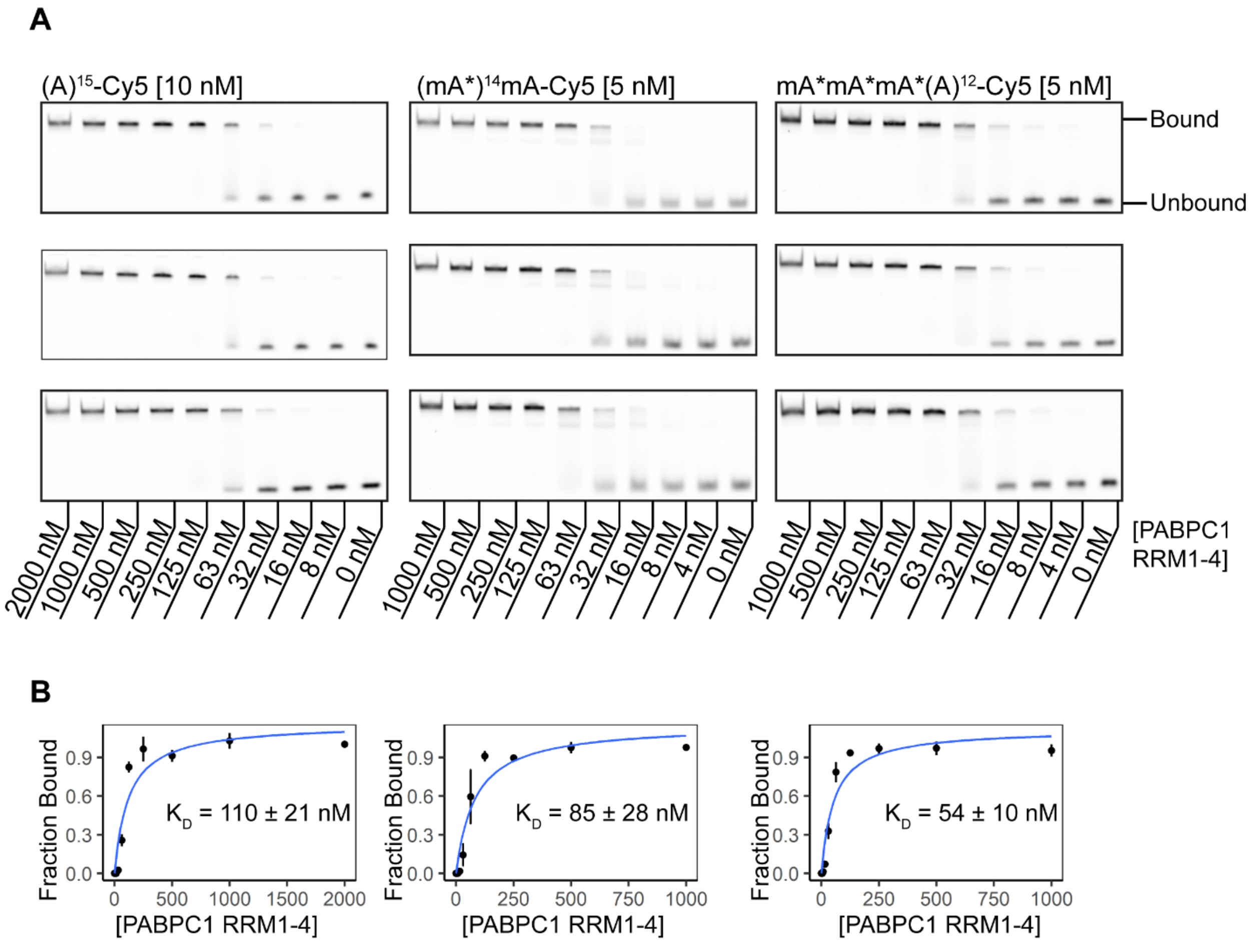

Figure S6: Modifications to adenosine bases do not disrupt PABPC binding to poly(A) RNA. **(A)** The binding affinity of the RNA-Recognition Motifs (RRMs) of human PABPC1 (PABPC) to unmodified and modified poly(A) RNAs 15nt in length were determined by Electrophoretic Mobility Shift Assay. PABPC at concentrations of 2000, 1000, 500, 250, 125, 63, 31, 16, 8, and 0 nM was incubated with unmodified poly(A) RNA at a concentration of 10 nM. PABPC at concentrations of 1000, 500, 250, 125, 63, 31, 16, 8, 4, and 0 nM were incubated with modified poly(A) RNA at a concentration of 5 nM. **(B)** Binding curves of PABPC to modified and unmodified poly(A) RNAs. The fraction of PABPC-bound RNA was plotted against the concentration of PABPC present. Binding curves and estimated Dissociation Constants (K_D) mean with standard deviation from three independent replicates are plotted in the graph.

Supplemental Tables:

Table S1: List and sequence of the different versions of Boosters

oJC#	Booster ID	version	Alias	Sequence
6480		1	Human LSM8-Booster1	TTrUrGrUrArCrArArArGrArUrUrUrArCrArGrArUrGrUrCrCrArArGrUrA
6285	CB3	1	CTNNB1-G1	TGrUrCrArGrGrCrArCrUrUrUrCrUrGrArGrArUrArCrCrArGrCrCrCrArC
6286	CB4	1	CTNNB1-G2	CTrUrCrArCrUrUrCrUrUrGrArGrUrCrArCrUrCrCrCrArArArArUrCrCrA
6287	CB5	1	CTNNB1-G3	ATrUrArGrArArArUrUrGrCrUrGrUrArGrCrArGrUrArUrUrCrArCrUrArU
6288	CB6	1	CTNNB1-G4	TGrArUrCrArArArArCrArUrGrArArArUrArGrArUrCrCrArCrCrUrGrC
6289	CB7	1	CTNNB1-G5	CTrCrUrUrGrArArGrCrArUrCrGrUrArUrCrArCrArGrCrArGrGrUrUrArC
7039	SB1	2	SynGAP-Booster1.1	AAAAAAAAAAAAAAAAAAAAAAAAAAAAAAAAAAAAAAAAAAAAAAAAAACACCCTGAAGTTGAAAGTTTGGAGGTGCC
7040	SB2	2	SynGAP-Booster1.3	AAAAAAAAAAAAAAAAAAAAAAAAAAAAAAAAAAAAAAAAAAAAAAAAAACACCCTGAAGTTGAAAGTTTGGAGGTGCCAAAAAAAAAAAAAAAAAAAAAAAAAAAAAAAAAAAAAAAAAAAAAAAAAA
7041	SB3	2	SynGAP-Booster2	AAAAAAAAAAAAAAAAAAAAAAAAAAAAAAAAAAAAAAAAAAAAAAAAAAACCTCAATTCCACCCCACCCAACAAAAAAAAAAAAAAAAAAAAAAAAAAAAAAAAAAAAAAAAAAAAAAAAAAA
7042	SB4	2	SynGAP-Booster3	AAAAAAAAAAAAAAAAAAAAAAAAAAAAAAAAAAAAAAAAAAAAAAAAAAGAAACACCGAAAAATCGTGGAGCTGAAAAAAAAAAAAAAAAAAAAAAAAAAAAAAAAAAAAAAAAAAAAAAAAAAA
7043	SB5	2	SynGAP-Booster4	AAAAAAAAAAAAAAAAAAAAAAAAAAAAAAAAAAAAAAAAAAAAAAAAAAATAAGTATAAATTTGGCCCCGGCAAAAAAAAAAAAAAAAAAAAAAAAAAAAAAAAAAAAAAAAAAAAAAAAAAA
7044	SB6	2	SynGAP-Booster5	AAAAAAAAAAAAAAAAAAAAAAAAAAAAAAAAAAAAAAAAAAAAAAAAAAAACCCCAGATAATTACAACAGCCAAAAAAAAAAAAAAAAAAAAAAAAAAAAAAAAAAAAAAAAAAAAAAAAAAA
6351	control	1	random guide 3	TTrArUrCrCrUrGrCrUrArCrGrGrArArGrCrUrCrArArUrCrArUrGrCrArU
7034	MB1	2	MeCp2-Mouse2/Human	AAAAAAAAAAAAAAAAAAAAAAAAAAAAAAAAAAAAAAAAAAAAAAAAAAGAGCCCACTTTAAAACAAGCGCAGGT
7033	MB2	2	MeCp2-Mouse1/Human	AAAAAAAAAAAAAAAAAAAAAAAAAAAAAAAAAAAAAAAAAAAAAAAAAAGAGCCCACTTTAAAACAAGCGCAGGTAAAAAAAAAAAAAAAAAAAAAAAAAAAAAAAAAAAAAAAAAAAAAAAAA
7035	CB1	2	CTNNB1-B1	AAAAAAAAAAAAAAAAAAAAAAAAAAAAAAAAAAAAAAAAAAAAAAAAAACACTTTCTGAGATACCAGCCCACAAAAAAAAAAAAAAAAAAAAAAAAAAAAAAAAAAAAAAAAAAAAAAAAA
7036	CB2	2	CTNNB1-B2	AAAAAAAAAAAAAAAAAAAAAAAAAAAAAAAAAAAAAAAAAAAAAAAAAAACTTTGGGATAAAGGCAACTGGAAAAAAAAAAAAAAAAAAAAAAAAAAAAAAAAAAAAAAAAAAAAAAAAAAA

7037	PB1	2	Pur-A-B1	AAAAAAAAAAAAAAAAAAAAAAAAAAAAAAAAAAAAAAAAAAAAAAAAAA TTCCACAATTGAATTGGCCACAAACAAAAAAAAAAAAAAAAAAAAAAAAA AAAAAAAAAAAAAAAAAAAAAAAAA
7038	PB2	2	Pur-A-B2	AAAAAAAAAAAAAAAAAAAAAAAAAAAAAAAAAAAAAAAAAAAAAAAAAA TAAACCAGATCTGAAAAGAGGCCTAAAAAAAAAAAAAAAAAAAAAAAAA AAAAAAAAAAAAAAAAAAAAAAAAA
6539	SB7	3	SynGAP-Booster3	mA*mA*mA*AAAAAAAAAAAAAAAAAAAAAAAAAAAAAAAAAAAAAAAAAA AAAAAAAAAAAAAAAAAAAAAAAAAAAAAAAAAAAAAAAAAAAAAAAAAA AAAAAGGACUGUGUGUCAGAGUAGGACAGAUC*mA*mA*mC
6540	CB7	3	CTNNB1 - Booster5	mA*mA*mA*AAAAAAAAAAAAAAAAAAAAAAAAAAAAAAAAAAAAAAAAAA AAAAAAAAAAAAAAAAAAAAAAAAAAAAAAAAAAAAAAAAAAAAAAAAAA AAAAACUCUUGAAGCAUCGUAUCACAGCAGGU*mU*mA*mC
6541	control	3	scramble RNA	mA*mA*mA*AAAAAAAAAAAAAAAAAAAAAAAAAAAAAAAAAAAAAAAAAA AAAAAAAAAAAAAAAAAAAAAAAAAAAAAAAAAAAAAAAAAAAAAAAAAA AAAAAUUAUCCUGCUACGGAAGCUCAAUCAUG*mC*mA*mU
7045	SB1-U	2	SynGAP 1-control-polu	TTTTTTTTTTTTTTTTTTTTTTTTTTTTTTTTTTTTTTTTTTTTTTTTTTCACC CTGAAGTTGAAAGTTTGGAGGTGCCTTTTTTTTTTTTTTTTTTTTTTTTTT TTTTTTTTTTTTTTTTTTTTTTTT
6483		3	pA15-Unmodified	5’- AAAAAAAAAAAAAAA-Cy5 -3’
6705		3	2'oMe_PSall_poly(A)15	mA*mA*mA*mA*mA*mA*mA*mA*mA*mA*mA*mA*mA*mA*mA/Cy5/
6706		3	2'oMe_PSthree_poly(A)15	mA*mA*mA*AAAAAAAAAAAA/Cy5/
6724		3	SynGAP Booster mod 75A	mA*mA*mA*AAAAAAAAAAAAAAAAAAAAAAAAAAAAAAAAAAAAAAAAAA AAAAAAAAAAAAAAAAAAAAAAAAAAAAAAGGACUGUGUGUCAGAGUA GGACAGAUC*mA*mA*mC
6725		3	SynGAP Booster mod 50A	mA*mA*mA*AAAAAAAAAAAAAAAAAAAAAAAAAAAAAAAAAAAAAAAAAA AAAAAAAAAAAAAAAAAAAAAAAAAAAAAAGGACUGUGUGUCAGAGUA GGACAGAUC*mA*mA*mC
6726		3	SynGAP Booster mod 25A	mA*mA*mA*AAAAAAAAAAAAAAAAAAAAAAGGACUGUGUGUCAGAGUA GGACAGAUC*mA*mA*mC
6727		3	SynGAP Booster mod 3A	mA*mA*mA*GGACUGUGUGUCAGAGUAGGACAGAUC*mA*mA*mC
oJC6352, oWH6361			Fluc booster+ PS+10A	TGCTCGAAGCGGCCGGCCGCCCCGA/iSp9/rArArArArArArArArArA
oJC6353 oWH6362			Fluc booster, no A, control	TGCTCGAAGCGGCCGGCCGCCCCGA

Table S2: List and the sequence of oligos used for PCR and qPCR analysis

oJC#	Alias	Sequence
6529	LSM8-forward	CGAGTATTCAGCTCTTCACAGGG
6530	LSM8-Reverse	CCCAAATCAAGCGCAGAATCTGT
6531	EN2-forward	GCTTGTCCTCTTTGTTCGGGTTC
6532	EN2-Reverse	CAACCTGGGCGCGCAGCCCAT
6544	DKK1-F	GGTATTCCAGAAGAACCACCTTG
6545	DKK1-R	CTTGGACCAGAAGTGTCTAGCAC
6546	C-myc-F	CCTGGTGCTCCATGAGGAGAC
6547	C-myc-R	CAGACTCTGACCTTTTGCCAGG
6638	Syngap1-F	CAACCTGCGAATGTGCTGTGAG
6639	Syngap1-R	CGCTGATAAGCCTGTCTGCGAT
6911	Syngap1 F (mouse)	CCGGACCAGCAGCTTTC
6912	Syngap1 R(mouse)	CCCAGGATGGAGCTGTG
6913	Actin F (mouse)	TCCTTCTGACCCATTCCCA
6914	Actin R (mouse)	TCCGGCATGTGCAAAGC
5544	Mecp2-F	TGAAGGCTGGACACGGAAGCTT
5545	Mecp2-R	CAGGGATGTGTCGCCTACCTTT
7046	PurA-F	CTCCTTGACTGTGGACAACAAG
7047	Pur-A-R	GCAGAAGGTGTGTCCGAACTTG
7048	CTNNB1-F	CAAGCAGAGTGCTGAAGGTGCTA
7049	CTNNB1-R	GATTCCTGAGAGTCCAAAGACAG
5067	Actin-F	CACCATTGGCAATGAGCGGTTC
5068	Actin-R	AGGTCTTTGCGGATGTCCACGT
6380	GAPDH-F	AATCCCATCACCATCTTCCA
6381	GAPDH-R	TGGACTCCACGACGTACTCA
7050	M13-F	TGTAAAACGACGGCCAGT
7051	M13-R	CACAGGAAACAGCTATGA
6730	SYN3 PROB	GTTGATCTGTCCTACTCTGACACACAGTC
7078	M.Mecp2F1	AGGAAGTCTGGCCGATCTG
7079	M.MeCp2R1	TCATTAGGGTCCAAGGAGGT
7080	M.Mecp2F2	GCTGGGATGTTAGGGCTCA
7081	M.Mecp2R2	CTGAAGGTTGTAGTGGCTCAT
7082	M-nfkb1-F	GCTGCCAAAGAAGGACACGACA
7083	M-nfkb1-R	GGCAGGCTATTGCTCATCACAG
7084	M-TNFa-F	GGTGCCTATGTCTCAGCCTCTT
7085	M-TNFa-R	GCCATAGAACTGATGAGAGGGAG

oJC6339, oWH6357	amplify off pWH231	GCTAATACGACTCACTATAGGTACTGTTGGTAAAG CCACCATGGAAG
oJC6340, oWH6358	amplify off pWH231	GGTTTGTCCAAACTCATCAATGTATCTTATCATGTC
oJC6341, oWH6359	amplify off pWH232	GCTAATACGACTCACTATAGGCTAGCCACC
oJC6342, oWH6360	amplify off pWH232	TTTTATTGCGGCCGCTTATTGTTCATTTTTGAGAAC TCGCTCAACGAA

Table S3: List of the plasmids

pJC#	Plasmid name	Description
315	pbluescript	backbone
1294	Mecp2 1tail-polyA	MB1
1295	Mecp2 2tail-polyA	MB2
1419	mouse mecp2-g1-2tail	MB3
1420	mouse mecp2-g2-2tail	MB4
1417	syngap1-1tail guide	SB1
1418	syngap1-2tail guide	SB2
1459	SynGAP1-Booster1-polU	SBU
1460	SynGAP1-PolA-booster2-PolA	SB3
1461	SynGAP1-PolA-booster3-PolA	SB4
1462	SynGAP1-PolA-booster4-PolA	SB5
1463	SynGAP1-PolA-booster5-PolA	SB6
1464	PurA-polA-boostert1-PolA	PB1
1465	PuaA-polA-booster2-polA	PB2
1466	CTNNB1-polA-booster1-polA	CB1
1467	CTNNB1-polA-booster2-polA	CB2
pJC1658, pWH231	pRL-SV40 (T7 promoter RLuc vector)	
pJC1659, pWH232	pGL4.10[luc2] (Fluc vector)	

Table S4: Formulation details and particle sizes for the LNPs

Code	Mol %				N/P Ratio	Z-Average (nm)	PDI
	Helper lipid	Dlin-MC3	Chol	DMG-PEG			
**C9 (brain)**	40	40	19.80	0.02	8	124.31 ± 2.93	0.21 ± 0.04
**FIII-7 (liver)**	5.45	54.55	39.92	0.08	4	132.23 ± 3.76	0.14 ± 0.03
