# Supplementary material for: Use of polyadenosine tail mimetics to enhance mRNA expression from genes associated with haploinsufficiency disorders
Source: Mol Ther Nucleic Acids. 2025 Jan 13;36(1):102453. doi: 10.1016/j.omtn.2025.102453 (PMC11834087; doi:10.1016/j.omtn.2025.102453)
Supplement: Document S2. Article plus supplemental information [file mmc2.pdf]

Molecular Therapy
Nucleic Acids
Original Article

# Use of polyadenosine tail mimetics to enhance mRNA expression from genes associated with haploinsufficiency disorders

Bahareh Torkzaban,[1] Yining Zhu,[3,6] Christian Lopez,[1] Jonathan M. Alexander,[5] Jingyao Ma,[3,6] Yongzhi Sun,[3,7] Katharine R. Maschhoff,[4] Wenqian Hu,[4] Michele H. Jacob,[5] Dingchang Lin,[3,7] Hai-Quan Mao,[3,6,7] Sophie Martin,[1] and Jeff Coller[1,2,3,6]

[1]Department of Molecular Biology and Genetics, Johns Hopkins University, Baltimore, MD 21205, USA; [2]RNA Innovation Center, Johns Hopkins University, Baltimore, MD 21218, USA; [3]Institute for NanoBioTechnology, Johns Hopkins University, Baltimore, MD 21218, USA; [4]Department of Biochemistry and Molecular Biology, Mayo Clinic, Rochester, MN 55905, USA; [5]Tufts University School of Medicine, 136 Harrison Avenue, Boston, MA 02111, USA; [6]Department of Biomedical Engineering, Johns Hopkins University School of Medicine, Baltimore, MD 21205, USA; [7]Department of Materials Science and Engineering, Johns Hopkins University, Baltimore, MD 21218, USA

**Polyadenosine (poly(A)) tails are nearly ubiquitous in human messenger RNA (mRNA) governing mRNA stability and translation. Crucially, the poly(A) tail regulates cytoplasmic gene expression by undergoing controlled removal upon exposure to the cytoplasm. Upon removal, mRNA ceases protein production and may subsequently be degraded or silenced. We have generated a therapeutic modality that tethers a poly(A) tail mimetic on the 3′ end of specifically targeted mRNAs, thereby enhancing their expression beyond their normal utility. This technology, which we term mRNA boosters, lends itself to uses on haploinsufficiency disorders, where reduced gene expression manifests in a disease state. By polyadenylating short RNA sequences antisense to the 3′ untranslated region (UTR) of specific mRNAs, we demonstrate that we can selectively and significantly enhance mRNA expression both *in vitro* and *in vivo*. We showcase the effectiveness of this technology on genes linked to autism spectrum disorders such as *SYNGAP1*, *MECP2*, *PURA*, and *CTNNB1*, illustrating increased expression in both human cell cultures and animal models. These findings indicate that small poly(A) tail mimetics can substantially enhance mRNA expression, providing the potential to efficaciously treat haploinsufficiency disorders.**

## INTRODUCTION

Haploinsufficiency, a genetic phenomenon, occurs when a single wild-type (WT) allele, along with a pathogenic variant allele, fails to produce adequate protein levels, resulting in the onset of disease.[1–3] With more than 300 identified human haploinsufficiencies documented to date, it is conceivable that there are numerous others yet to be characterized. The challenges presented by these conditions to disease-modifying technologies are significant, primarily due to their etiology, which hinges on the precise amount of protein required for normal physiological function.

Many haploinsufficiencies stem from spontaneous germline mutations, such as those observed in Dravet syndrome.[4] These instances underscore the complexity of addressing such genetic disorders. In the context of haploinsufficiencies originating from sporadic mutations, there arises a pressing need for mutation-agnostic strategies that augment protein expression from the WT allele. Such approaches could potentially offer promising avenues for intervention and treatment.

In the realm of treating haploinsufficiencies, there is growing interest in exploring the potential of directly targeting endogenous transcripts as a therapeutic strategy.[5–9] While changes in gene expression are often attributed to programmed transcriptional variability, it is essential to recognize that substantial regulation of mRNA expression also takes place within the cytosol.[10–12] Indeed, post-transcriptional regulation plays a pivotal role in mRNA stabilization and translation kinetics. This intricate process involves a variety of sequence and structural elements within mRNA molecules that recruit specific factors, either enhancing or inhibiting their activity.[13–15] Each transcript undergoes degradation and translation at its unique rate, finely regulated by cellular mechanisms. These regulatory mechanisms exert significant influence on the protein output per transcript, thus presenting an opportunity for therapeutic intervention. Understanding and manipulating these processes could provide avenues for effectively addressing haploinsufficiencies and related genetic disorders.

Arguably, the critical feature that influences the cytoplasmic expression of mRNAs is the 3′ polyadenosine (poly(A)) tail and its associated protein, poly(A)-binding protein (PABPC1).[12,16] Nearly all

Received 8 August 2024; accepted 10 January 2025;
https://doi.org/10.1016/j.omtn.2025.102453.

**Correspondence:** Jeff Coller, Department of Molecular Biology and Genetics, Johns Hopkins University, Baltimore, Maryland 21205, USA.
**E-mail:** jmcoller@jhmi.edu


Published by Elsevier Inc. on behalf of The American Society of Gene and Cell Therapy.
This is an open access article under the CC BY license (http://creativecommons.org/licenses/by/4.0/).

eukaryotic mRNAs, except certain mammalian histone transcripts, bear poly(A) tails on their 3′ end. These tails are added during transcription in the nucleus and are indispensable for the expression of mature mRNAs in the cytoplasm. Typically, poly(A) tails exhibit a relatively uniform length, approximately 150–200 nucleotides in mammals[17] and 60–70 nucleotides in yeast.[18] PABPC1 binds to 12 adenosine residues but protects 25, thus a typical poly(A) tail of 200 nucleotides has eight molecules of PABPC1 bound. It is crucial to note that poly(A) tails are not static; they undergo dynamic changes in length in response to specific cellular signals once in the cytoplasm.[19–21]

The poly(A) tail and PABPC1 play a dual role in modulating mRNA fate, impacting both translational status and stability. They act in concert with the 7-methylguanosine cap (m7Gppp) on the 5' end of mRNA to enhance translation.[22–24] Consequently, shortening of the poly(A) tail (deadenylation) can dampen translation while also facilitating de-capping and subsequent mRNA decay. The rate of deadenylation is transcript specific and can be finely regulated in response to cellular cues, enabling the targeted repression or degradation of specific transcripts via eviction of PABPC1. Moreover, in certain circumstances, poly(A) tails can be elongated in the cytoplasm to revive translationally repressed transcripts or uphold their stability.[25–28] The dynamic nature of poly(A) tails thus plays a pivotal role in governing gene expression, exerting profound impacts across various realms of eukaryotic biology, including early development, the inflammatory response, and synaptic plasticity.[29–31]

Here, we introduce an innovative approach designed to boost the expression of targeted mRNAs. Our method involves using antisense oligonucleotide guide sequences that are polyadenylated to mimic the natural poly(A) tail of the transcript and increase PABPC1 binding, thereby enhancing its expression. We demonstrate effectiveness in controlling both reporter genes and endogenous transcripts in human cell culture and mice. Crucially, we demonstrate that mRNA boosters can elevate the expression of mRNAs associated with dosage disorders, including *MECP2*, *CTNNB1*, *PURA*, and *SYNGAP1*. Additionally, we demonstrate that poly(A) tail mimetic technology can be optimized using industry standard chemical modifications to enhance durability and efficacy. We hypothesize that poly(A) tail mimetics is a promising technology that could result in disease-modifying treatment options for patients living with haploinsufficiency disorders that currently lack treatment options.

## RESULTS

### Design, synthesis, and screening of mRNA boosters

Previous studies have demonstrated that a poly(A) tail or its associated protein (PABPC1) can be delivered to the 3′ untranslated region (UTR) of an mRNA via exogenous means and it will stimulate gene expression via augmented stability and translation.[32] Pioneering research by Sheets and Wickens (1995)[33] demonstrated that providing a poly(A) tail *in trans* could effectively enhance the translation of the c-mos transcript in stage IV *Xenopus* oocytes. Building upon this work, we designed a simple and facile means to tether a poly(A) tail and its associated activities to any mRNA. Using a variety of approaches, we synthesized small oligonucleotide sequences that were antisense to the 3′ UTR of target genes (we term these guide sequences). We screened and selected optimal guide sequences based upon the computational approached developed by Sanjana's group.[34] The highest-scoring guide sequences were each 22–30 nucleotides. Guide sequences were then polyadenylated using either enzymatic approaches or synthetic approaches, showing the plasticity of the system (see materials and methods; Table S1; and Figure 1A). Briefly, as a facile means to screen through multiple guide sequences, they were first chemically synthesized as oligonucleotides then polyadenylated in 3′ *in vitro* using *Escherichia coli* poly(A) polymerase, resulting in a poly(A) tail of approximately 150 nucleotides (version 1.0) (Figure S1A). We also reasoned that positioning the poly(A) sequence at the 5′ end of the guide sequence would prevent the natural action of the CCR4/CAF1 deadenylase, which shortens tails via a 3′ to 5′ exonucleolytic action. We therefore designed boosters with a 50-nucleotide poly(A) tail in 5′ or both 5′ and 3' (version 2.1 and version 2.2, respectively) that was generated by *in vitro* transcription (IVT). These strategies (explained in detail below and in the materials and methods) were used throughout the manuscript and led to similar results. Lastly, an improved version of the booster (version 3) was completely chemically synthesized and comprised modified nucleotides (Figure 1A).

### mRNA boosters enhance mRNA expression *in vitro*

In our initial experiments, we co-transfected mRNA reporters for Firefly and Renilla into HEK293 cells along with oligonucleotides targeting the Firefly mRNA's 3′ UTR in an antisense manner. The specific oligonucleotides used either lacked a poly(A) tail, contained a 10-nucleotide tail added chemically, or possessed a ∼200-nucleotide tail added enzymatically. As illustrated in Figure 1B, we observed a remarkable 6-fold increase in Firefly mRNA expression when annealed to the oligonucleotide carrying a 200-nucleotide poly(A) tail and transfected to the cells. No significant enhancement was observed in Renilla mRNA expression, nor in the control mRNA GAPDH. These data argue that a mRNA Booster can dramatically enhance the expression of an *in vitro* transcribed mRNA when co-transfected.

Next, we aimed to target an endogenous mRNA to assess the feasibility of our approach in modulating cellular gene expression. The *LSM8* mRNA is a small, ubiquitously expressed transcript whose mRNA stability is known to be tightly regulated post-transcriptionally.[35,36] To target *LSM8* mRNA, we designed antisense guide RNAs directed against its 3′ UTR and polyadenylated them using enzymatic means. As a control, we generated a second RNA oligo, which was computationally demonstrated to be devoid of genomic and transcriptome interactions (Table S1). The control oligo was also polyadenylated to the same extent as the *LSM8* oligo using poly(A) polymerase. Subsequently, the polyadenylated oligos were transfected into HEK293 cells at a concentration of 20 nM using lipofectamine. A working concentration of 20 nM was selected based on an initial dose curve which exhibited a 3-fold increase in *LSM8*

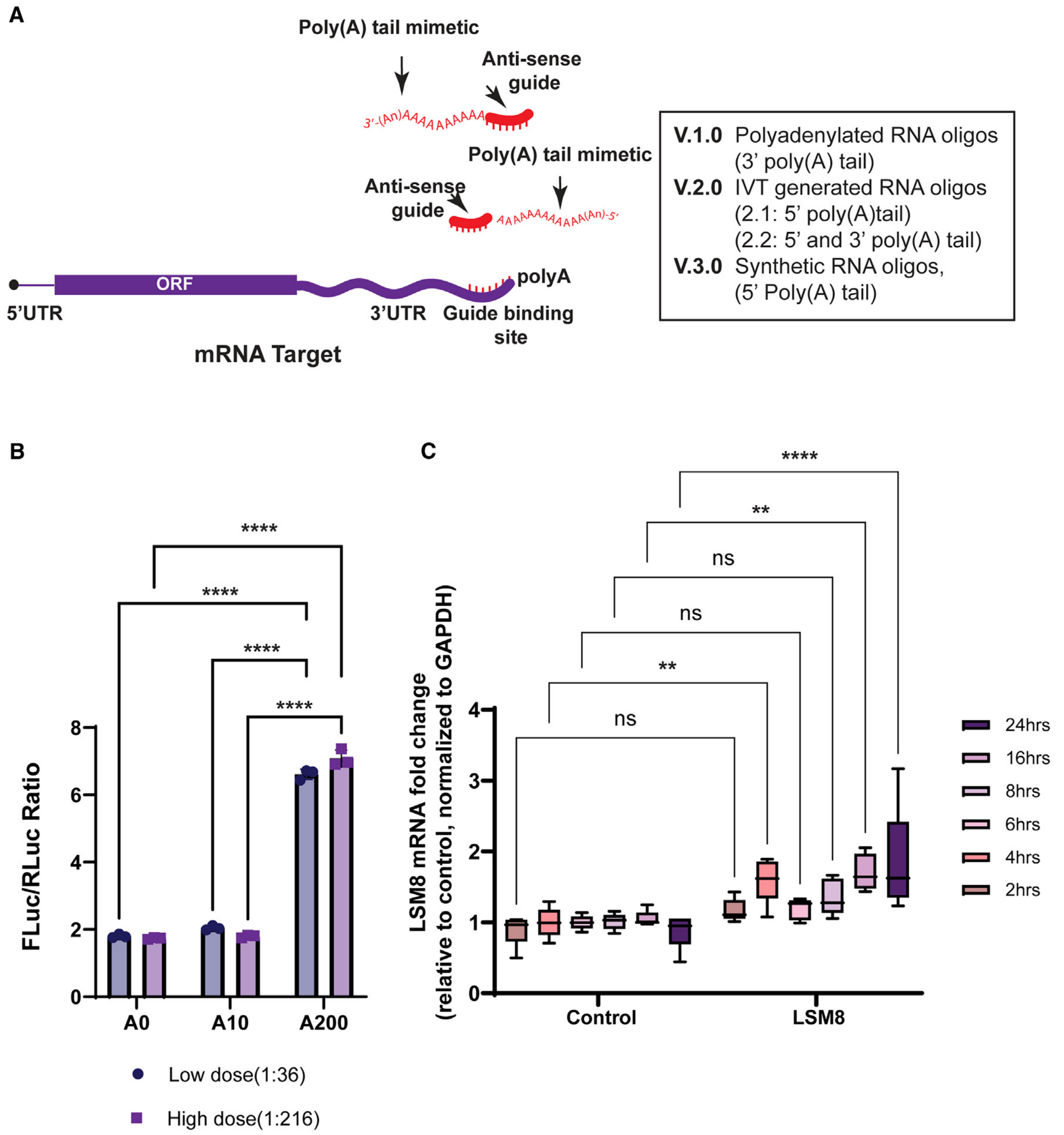


**Figure 1. Poly(A) tail mimetics enhance the expression of target mRNAs**

(A) Schematic of the basic design of the mRNA booster: a 30-nucleotide sequence complementary to a particular region of the 3′ UTR of a targeted mRNA, which bears a poly(A) tail. (B) A poly(A) mimetic enhances the expression of an *in vitro* transcribed Firefly luciferase reporter in HEK293 cells, with a significant (one-way ANOVA, ****$p < 0.00001$) 6-fold increase in FLuc over Renilla activity when the booster bears a 200-nucleotide poly(A) tail, 48 h after transfection. The results are depicted for a booster without a poly(A) tail, with a short 10-nucleotide tail, or a long 200-nucleotide tail; for two different FLuc mRNA to booster ratios (low dose, 1:36; and high dose, 1:216). (C) Targeting an endogenous mRNA confirms the booster efficiency to enhance cellular gene expression. *LSM8* mRNA levels normalized to *GAPDH* are represented, after treatment with 20 nM final concentration of LSM8- specific booster compared to a non-specific polyadenylated control. Cells were harvested 2, 4, 6, 8, 16, and 24 h after transfection. The comparison between control vs. booster treated in each time point indicates significant increase (**) after 4-, 16- and 24-h incubation. Two-way ANOVA, **$p < 0.001$, ****$p < 0.00001$.

mRNA levels 16 h after incubation (Figure S1B). Cells were harvested at various time points within 24 h (between 2 and 24 h) following transfection, and mRNA expression was investigated using quantitative reverse-transcription PCR qRT-PCR. Primer pairs specific to *LSM8* were validated to ensure linearity and sensitivity, capable of detecting 2-fold variations in *LSM8* mRNA levels (Figure S1C).

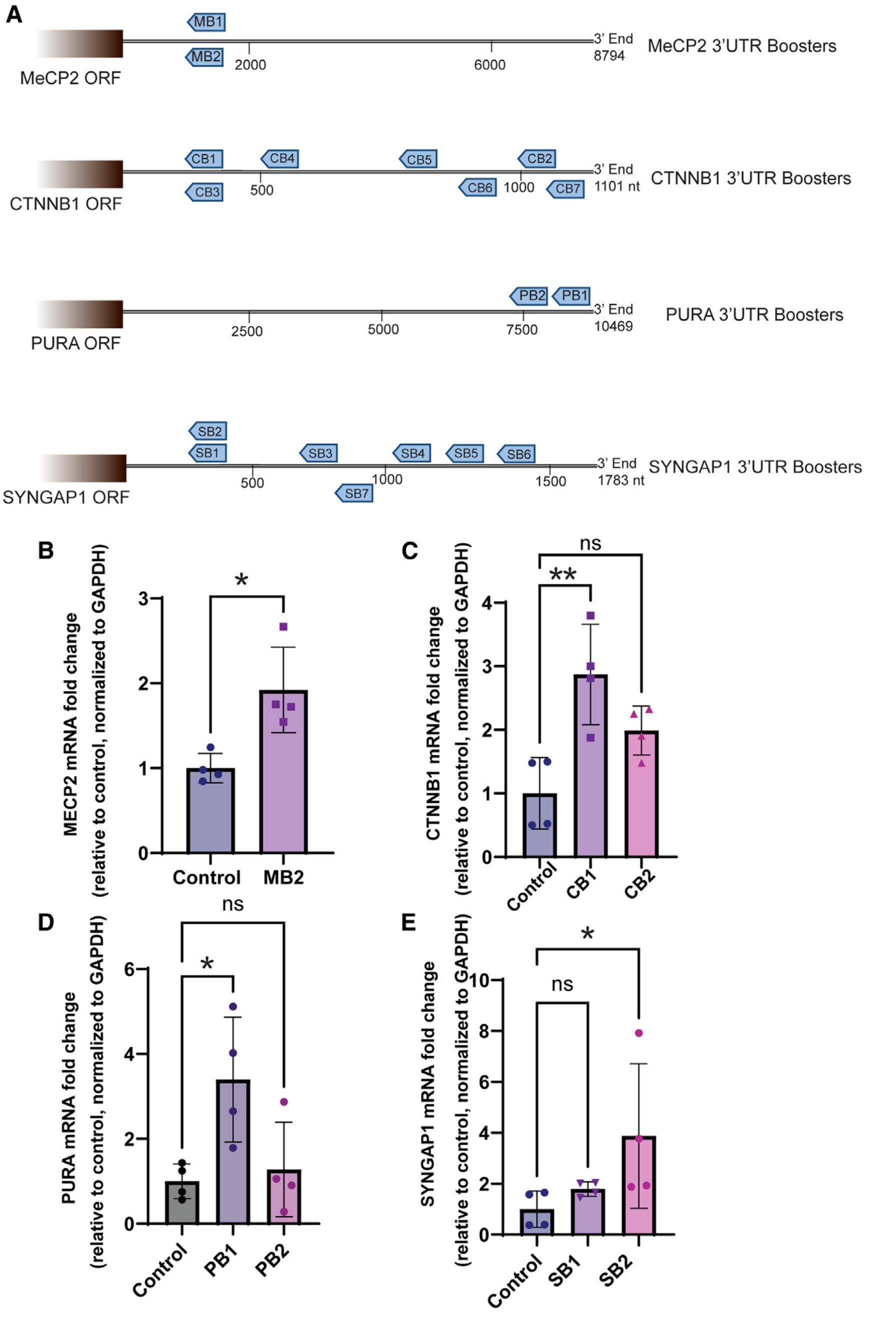


**Figure 2. mRNA boosters enhance haploinsufficiency-associated mRNAs**

(A) Schematic of the guide sequences position on the 3′ UTR of their target genes: *MECP2*, *CTNNB1*, *PURA*, and *SYNGAP1* mRNAs, used throughout the study. Guide sequences spanning distinct regions were evaluated and assigned. (B) Levels of *MECP2* mRNA measured by qRT-PCR in SH-SY5Y cells transfected with 40 nM booster V.2.2 (MB2) against 3′ UTR of the *MECP2* or a control booster not targeting *MECP2* (control) and harvested 16 h after transfection. Welch's t test, $^*p = 0.05$. (C and D) Levels of *CTNNB1* (C) and *PURA* (D) mRNAs measured by qRT-PCR in HEK293 cells transfected with boosters V.2.2 targeting distinct regions of the 3′ UTR (B1 and B2) or a non-specific control (control), 24 h after transfection. Ordinary one-way ANOVA, $^*p = 0.05$, $^{**}p = 0.005$. (E) *SYNGAP1* mRNA levels measured by qRT-PCR in SH-SY5Y cells transfected with two versions of booster V.2.0 (SB1 and SB2) targeting *SYNGAP1* mRNA 3′ UTR or a non-specific control (control). SB2 showed a significant (*) up to 4-fold increase in the mRNA level. Welch's t test, $^*p = 0.05$.

As shown in Figure 1C, transfection of the polyadenylated guides directed against *LSM8* led to a time-dependent increase in mRNA abundance, with over 1.5-fold difference relative to control observed by 16–24 h post transfection. In contrast, the control polyadenylated oligo showed no discernible effect on *LSM8* mRNA levels (Figure S1D). These findings collectively demonstrate the ability to supply a poly(A) tail *in trans*, resulting in the augmentation of both *in vivo*- and *in vitro*-derived mRNAs. This underscores the potential of our approach in effectively modulating cellular gene expression.

### mRNA boosters enhance the expression of mRNAs associated with haploinsufficiency disorders

In our preliminary data, we demonstrated that our mRNA booster technology can be used to augment the expression of both transiently expressed and endogenous mRNAs. We next turned our attention to testing booster technology on mRNAs associated with known haploinsufficiencies. Haploinsufficiency disorders arise when one of the two alleles is non-functional, resulting in insufficient expression of the protein necessary for normal health. Many human haploinsufficiencies manifest as developmental encephalopathies, typically emerging in infancy or childhood and characterized by frequent seizures of various types, intellectual disability, and substantial developmental delay, regression, or plateau.[37–42] We chose to focus on the *MECP2*, *CTNNB1*, *PURA*, and *SYNGAP1* mRNAs as proof of concept for the mRNA booster technology. For each target, guide sequences spanning distinct regions were evaluated and assigned (refer to materials and methods for details) (Figure 2A). mRNA abundance was assessed using real-time qRT-PCR, employing primer pairs validated to yield linear results and capable of detecting 2-fold differences in expression. Additionally, control guide sequences lacking specificity to human mRNAs but possessing polyadenylation were employed (Table S1). As illustrated in Figure 2B, our findings indicate that the booster directed against *MECP2* mRNA led to a 2-fold increase in mRNA abundance. Furthermore, boosters exhibited variable

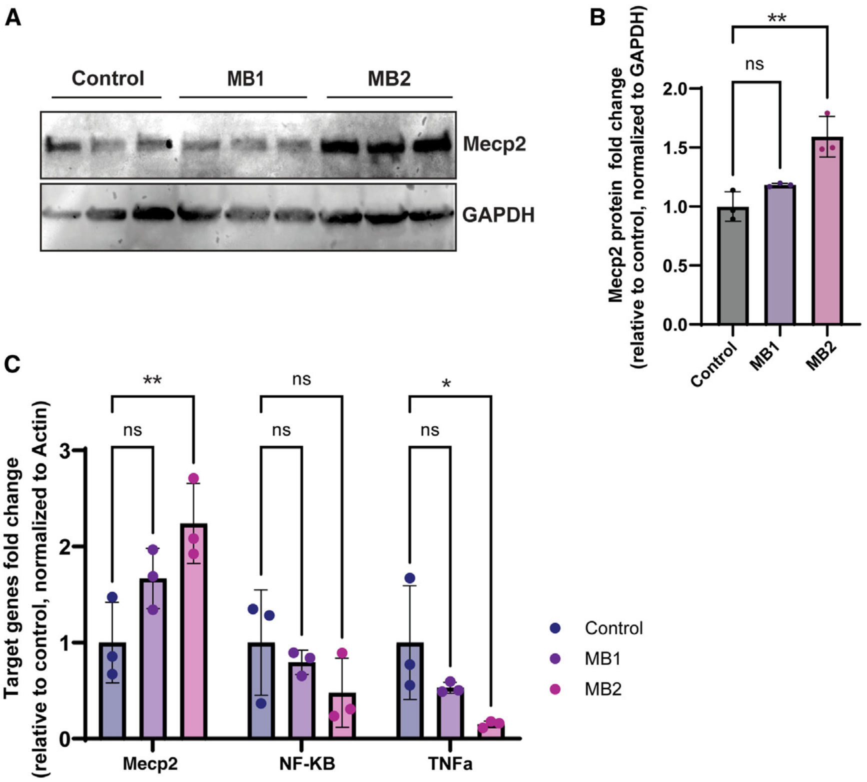


**Figure 3. Booster technology enhances *Mecp2* expression *in vivo***

(A) Western blot for *Mecp2* from liver lysates of 6-week-old mice injected with 25 μg of LNP-encapsulated boosters (version 2.0, MB1 and MB2, or non-specific control) by tail vein injection. The tissues were collected 48 h after injection. (B) Quantitation of the western blot in (A), showing a significant (**) 1.5-fold increase in protein levels in the animals injected with MB2 compared to the control. Ordinary one-way ANOVA, $^{*}p$ = 0.05, $^{**}p$ = 0.005. (C) *Mecp2* mRNA levels from liver lysates of mice injected with LNP-encapsulated boosters (version V.2, MB1 and MB2, or non-specific control) by tail vein injection as in (A) and (B), measured by qRT-PCR. In agreement with the protein levels, there is a significant (**) 2-fold increase in the level of *Mecp2* mRNA in mouse liver. The mRNA levels of a downstream gene of *Mecp2* (*TNF-α*) along with an inflammatory marker (*NF-κB*) are measured by qRT-PCR. One -way ANOVA,$^{*}p$ = 0.05, $^{**}p$ = 0.001.

effects on *CTNNB1* mRNA expression, as demonstrated in Figure 2C. Notably, *PURA* and *SYNGAP1* mRNAs exhibited the most substantial increases in expression, with gains of between 3- and 4-fold observed (Figures 2D and 2E). These results collectively highlight the capacity of boosters to selectively modulate the expression of genes associated with haploinsufficiency disorders *in vitro* at the mRNA level.

### Booster technology augments Mecp2 expression in WT mice

We next tested the efficacy of mRNA boosters in mice starting with methyl CpG binding protein 2 (*Mecp2*). MECP2 is a ubiquitously expressed protein but appears to be essential for the normal function of nerve cells.[43,44] In particular, alteration of the *MECP2* gene results in Rett syndrome; a rare genetic neurological and developmental disorder that causes a progressive loss of motor skills and language.[45] Human and mouse *MECP2* 3′ UTRs are about 80% conserved. We developed boosters to target both human and mice, using IVT (MB1 and MB2 based on version 2, refer to materials and methods for details). The mouse-specific boosters were injected as a lipid nanoparticle (LNP)/booster formation into WT mice via high-pressure tail vein injection. The employed FIII-7 LNPs (Table S4) in this study were selected based on a wide-range screening of 1,080 LNPs.[46] After approximately 48 h post injection, we harvested and processed liver samples for evaluation of MECP2 expression at both the mRNA and protein levels. Notably, booster MB2 elicited a significant 1.5-fold increase in Mecp2 protein levels *in vivo* (Figures 3A and B and S2) accompanied by a more than 2-fold increase in *Mecp2* mRNA levels (Figure 3C). These results are fully consistent with the observed effects for these boosters *in vitro* (Figures 2B vs. 3C). Thus, booster technology can augment *MECP2* gene expression both *in vitro* and *in vivo*. It should be noted, however, that, given the X-linked nature of the *MECP2* gene, it is not known if the booster technology would be a feasible approach for treating *MECP2*-related disorders. Interestingly, treatment with MECP2 booster did not lead to an increase in the mRNA level of mediators of inflammation such as nuclear factor κB (*NF-κB*) or tumor necrosis factor alpha (*Tnf-α*) in the same liver samples, suggesting it does not trigger general inflammation in the mice within 48 h (Figure 3C). *Tnf-α* mRNA levels were even decreased when *Mecp2* levels were increased, which can be due to downstream effects on genes likely to be affected by *Mecp2* function as a transcriptional regulator.[47,48]

### Booster technology augments *CTNNB1* expression and function in different cell types

CTNNB1 neurodevelopmental disorder is characterized in all individuals by mild-to-profound cognitive impairment.[49–51] The *CTNNB1* gene encodes the β-catenin protein, which has dual functions in regulation and coordination of cell-cell adhesion and Wnt-responsive gene transcription. mRNA boosters targeting *CTNNB1* mRNA were developed (materials and methods) and subjected to thorough testing *in vitro*, *in vivo*, and in human induced pluripotent stem cell (iPSC)-derived neurons.

First, multiple boosters spanning distinct regions of the *CTNNB1* 3′ UTR were assessed for a dose response in HEK293 cells (Figure 4A and S3). The *CTNNB1* 3′ UTR spans 1,101 nucleotides. Five distinct polyadenylated guides targeting positions 376, 492, 708, 892, and 1,024 nucleotides (relative to the *CTNNB1* stop codon) were chosen

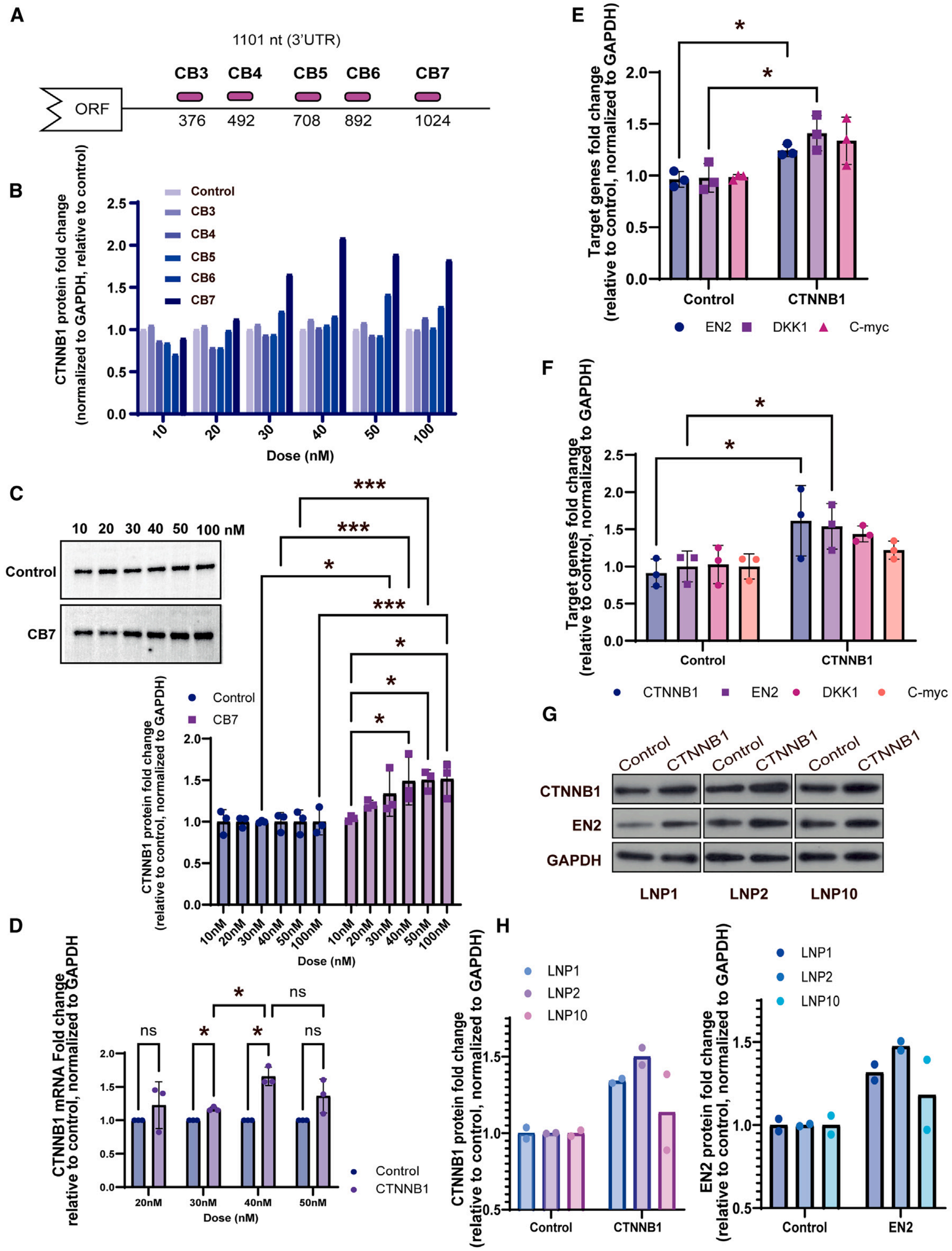


**Figure 4. Poly(A)-tail mimetics increase *CTNNB1* expression in different cell types**

(A) Schematic of five distinct boosters along *CTNNB1* 3′ UTR, designed between nucleotide 376 and 1024. (B) The bar graph presents the result of quantified western blotting (Figure S3) for *CTNNB1*, showing the position and dose-dependent efficiency of the boosters targeting *CTNNB1* compared to a non-specific booster control. To screen and select the most effective booster for *CTNNB1*, HEK293-STF cells were transfected with different doses (10, 20, 30, 40, 50, and 100 nM) of five distinct *CTNNB1* boosters

*(legend continued on next page)*

because they were 95%–100% conserved between mice and humans. Boosters positioned between 892 and 1,052 nucleotides downstream of the stop codon exerted the most pronounced effect on *CTNNB1* expression (Figures 4B and S3). These differences may reflect a variety of factors, including binding strength and accessibility within the native transcript (i.e., regions free of protein factors or RNA secondary structure). Western blot screening revealed a position and dose-dependent response in *CTNNB1* expression in cell culture, reaching a plateau at approximately 40 nM booster CB7, located at 1,024 nucleotides (Figures 4A–4C and S3). This finding was also confirmed by qPCR analysis (Figure 4D).

CTNNB1 is a critical regulator of the Wnt signaling pathway.[52] To verify that booster stimulation of CTNNB1 results in functional expression, we monitored the transcription of downstream Wnt targets[52–55] in HEK293 cells (Figure 4E), SH-SY5Y cells (Figure 4F), and in cultured human *CTNNB1* heterozygote iPSC-derived neurons (Figures 4G and 4H). Importantly, we observed a concomitant increase in *CTNNB1* expression and its associated Wnt targets, *EN2*, and *DKK1* when CTNNB1 booster was transfected. These data demonstrate that booster technology results in normal target engagement for CTNNB1. These findings underscore the efficacy and versatility of mRNA boosters in modulating gene expression across different cellular contexts.

### Booster technology augments *SYNGAP1* expression in the brain

SYNGAP1 deficiency features developmental delay or intellectual disability, along with generalized epilepsy, autism spectrum disorder, and other behavioral abnormalities.[38,56–58] This rare genetic disorder results from mutations or deletions in the *SYNGAP1* gene, responsible for encoding the synaptic Ras GTPase-activating protein-1.[57,59]

We investigated the potential of the mRNA booster approach to enhance *SYNGAP1* expression using various strategies. The *SYNGAP1* 3′ UTR spans 1,783 nucleotides and exhibits an 82.88% sequence identity between humans and mice. To assess whether the position within the 3′ UTR influences booster efficacy, we designed polyadenylated guides targeting different regions of the 3′ UTR (Figure 5A). Our findings indicate that boosters generally augmented *SYNGAP1* mRNA abundance in SH-SY5Y cells, with those positioned between 384 and 1,078 nucleotides downstream of the stop codon exerting the most pronounced effect on expression (Figure 5B). Together, these data highlight that position effects are idiosyncratic to individual mRNAs as these findings are distinct for those observed for *CTNNB1* (where proximity toward the 3′ end appeared to be most important). Again, these data most likely reflect a number of possibilities, including sequence accessibility, structure, and binding efficiency.

In contrast to *MECP2* and *CTNNB1*, which are expressed ubiquitously, *SYNGAP1* expression is restricted to the brain and found in the hippocampus, cerebral cortex, and midbrain, but poorly expressed in the cerebellum.[60,61] In order to test whether booster technology can augment *SYNGAP1* expression in the brain, we performed an intracranial injection experiment. In brief, this technology was used to inject LNPs directly into hippocampus using a flexible and stretchable nanoneedle with a nanoscale thickness that can be implanted by minimally invasive injection through a polymeric catheter.[46] Boosters were encapsulated in a LNP formulation designed with neuronal tropism (Figure S4; Table S4). These boosters were directly administered into the mouse hippocampus at a dose of 25 μg (RNA). Following a perfusion period of 48 h, brain samples were collected and subjected to analysis to evaluate Syngap1 expression levels. In a limited animal study ($n = 3$), we systematically examined Syngap1 expression in key brain regions, including the hippocampus, cerebral cortex, cerebellum, and midbrain. Notably, our preliminary results on three mice (one injected with scrambled RNA and the other two animals injected with booster SB1 and SB2) revealed a 2- to 3-fold increase in Syngap1 protein and 1.5-fold mRNA expression attributed to booster RNAs compared to control samples (Figures 5C and S5). Importantly, these effects were not confined solely to the hippocampus but were also evident in other brain regions, except for the cerebellum, where *Syngap1* expression is minimal (Figures 5C and 5D). To evaluate the significance of these observations, we conducted additional experiments on 16 additional mice, maintaining an approximate 50% male-to-female ratio. Notably, when focusing specifically on the hippocampus, we observed a notable 1.5- to 2-fold increase in both Syngap1 mRNA and protein expression levels (Figures 5E, 5F, and –5G). These robust findings collectively underscore the efficacy of RNA booster technology in augmenting SYNGAP1 expression within the brain.

### Chemically modified mRNA boosters exhibit durability and efficacy

In order to improve the overall feasibility of the RNA booster technology as a potential therapeutic modality, we employed a chemical synthesis protocol and incorporated unique nucleotide modifications known to improve the overall efficacy and durability of nucleic acid therapeutic approaches.

V.1.0 (CB3, CB4, CB5, CB6, and CB7). Booster CB7 increases the protein level up to 2-fold compared to control at 40 nM booster or higher. (C) Representative western blotting and quantifications for three biological replicates showing the significant dose-dependent increase of CTNNB1 protein levels upon exposure to booster CB7 compared to control (for more blots and loading control, see Figure S3). Two-way ANOVA, $^{*}p = 0.05$, $^{***}p = 0.0005$. (D) qRT-PCR analysis confirms booster CB7 efficiency to increase *CTNNB1* mRNA levels in a dose-dependent manner. Two-way ANOVA, $^{*}p = 0.01$. (E and F) mRNA levels of Wnt signaling pathway markers measured by qRT-PCR, illustrating the functional enhancement of CTNNB1 following the increase in CTNNB1 expression, in two different cell lines: HEK293-STF (E) and SH-SY5Y (F) cells. Welch's t test, $^{*}p < 0.05$. (G and H) Protein levels of B-catenin and its downstream effector *EN2* in β-catenin heterozygote, human iPSC-derived neurons, 48 h after transfection with LNP-packed boosters against *CTNNB1* (CB7) or a non-specific control. Three different LNP formulations were tested (LNP1, LNP2, and LNP10; see Figure S2). (G) A representative western blot and (H) a quantitation of CTNNB1 and EN2 from western blots from two biological replicates.

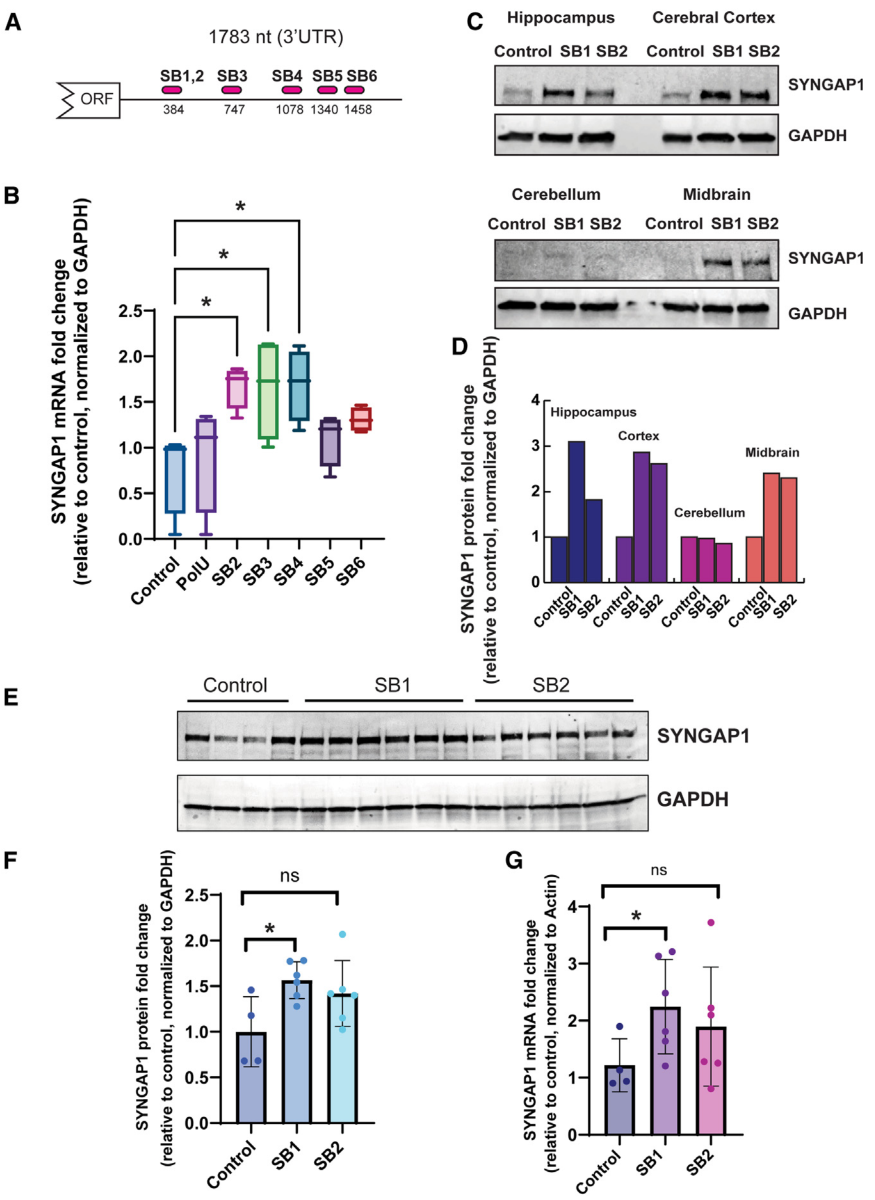


**Figure 5. Booster technology enhances *SYNGAP1* expression in the brain**

(A) Screening 3′ UTR of *SYNGAP1* for optimal boosting activity using Booster V.2.2. The schematic shows five distinct oligos targeting the 3′ UTR of *SYNGAP1*mRNA. (B) qRT-PCR analysis indicated that the booster position influences its efficiency in enhancing gene expression. SH-SY5Y cells were transfected with 40 nM boosters (SB2, SB3, SB4, SB5, SB6), an oligo with the same sequence as SB1,2 which poly(A) tail replaced with poly(U) tail, and a non-specific scrambled booster. Boosters SB3 (nucleotide 747) and SB4 (nucleotide 1078) produced the greatest increase in *SYNGAP1* mRNA levels relative to the scrambled control (median 1.8-fold). Two-way ANOVA, $^*p = 0.05$. (C and D) Detection of SYNGAP1 by Western blotting in different brain regions of 6-week-old mice. Brain tissue harvested 48 hrs after injection with LNP-encapsulated SynGAP1 Booster V.2.0 (SB1 and SB2) or a non-specific control Booster, into the mice left hippocampus. (D) Quantification of Syngap1 from (C) normalized to GAPDH, expressed as a ratio to the control treatment. The western blot showed an increase in Syngap1 levels in the hippocampus, cerebral cortex, and midbrain; however, Syngap1 expression was not detectable in the cerebellum. (E and F) Protein levels of Syngap1 in the hippocampus of mice injected with 25 μg of LNP-packaged Syngap1 boosters V.2.0 (SB1 and SB2) or a non-specific scrambled control, measured by western blot. n = 4 control (scrambled RNA-treated mice), n =6 SB1-treated mice, and n = 6 SB2-treated mice. (F) Quantification of (E) (SYNGAP1 signal normalized to GAPDH, expressed as a fold change over the average in the control). (G) *Syngap1* mRNA levels measured by qRT-PCR on samples from the same animals as in (E) and (F), confirming a *Syngap1* expression enhancement of up to 3-fold after treatment with SYNGAP1-specific boosters compared to control. One-way ANOVA, $^*p < 0.01$.

We started with a completely chemically synthesized booster of 130 nucleotides in length, comprising 100 riboadenosines fused to the 5′ end of the 30-nucleotide guide sequence (version V3.0). Positioning the poly(A) sequence at the 5′ end of the guide sequence was deliberate to prevent the natural action of the CCR4/CAF1 deadenylase, which shortens tails via a 3′ to 5′ exonucleolytic action.[62,63] Moreover, both 5′ and 3′ ends were modified with 2′-O-methyl and phosphorothioate (PS) linkages to prevent promiscuous exonuclease activity (Figures 6A and 6B). First, we tested the efficiency of PABPC1 binding to chemically synthesized/modified boosters. Using electrophoretic mobility shift assays (EMSAs) we measured dissociation constants between modified poly(A) sequences 15 nucleotides in length and recombinant PABPC1. As shown, EMSA analysis indicated that modifications of adenosine bases do not disrupt *in vitro* PABPC1 binding to poly(A) RNA, with a mean dissociation constant ($K_D$) of unmodified vs. fully modified vs. first three nucleotides modified (3NT Mod) of 110 vs. 85 vs. 54 nM, respectively (Figures 6C and S6). Next, we transfected chemically modified boosters (V.3.0) into SH-SY5Y cells and monitored *SYNGAP1* mRNA levels by qRT-PCR. Importantly, chemically modified boosters delivered a highly consistent 1.6-fold increase in *SYNGAP1* mRNA level in SH-SY5Y cells (Figure 6D). Unmodified boosters do not show any appreciable influence on *SYNGAP1* mRNA expression 7 days post transfection (in all earlier *in vitro* studies, cells were harvested 24–48 h post transfection). Consistently, we observed a marked increase in durability of these chemically modified boosters. Specifically, we transfected SH-SY5Y cells with modified or unmodified boosters, washed the medium, and incubated cells for 7 days. Cells were then harvested, RNA extracted, and northern

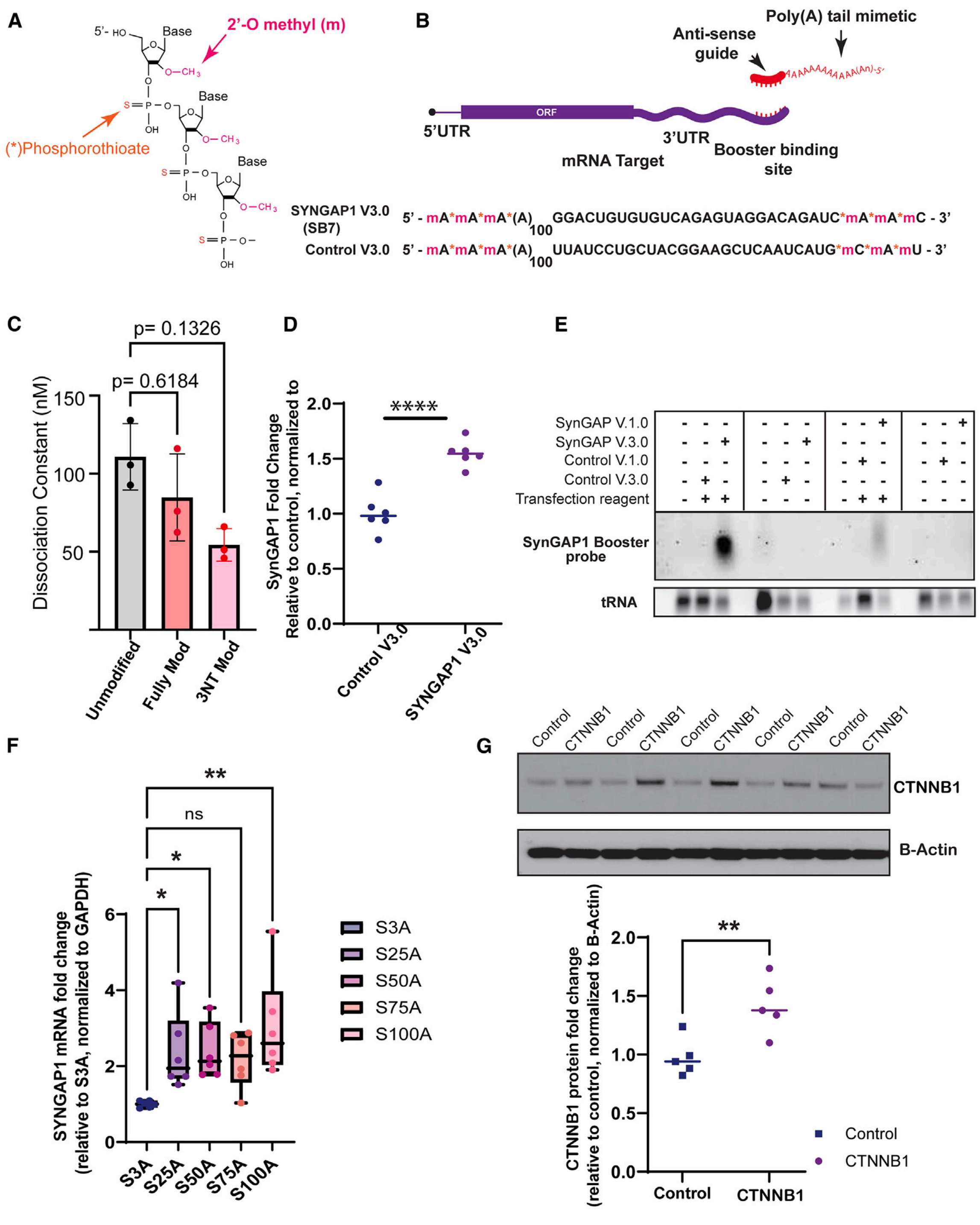


**Figure 6. Optimization of mRNA Booster to enhance stimulatory activity**

(A) Chemical structure of the modifications (phosphorothioate and 2′-O-methyl) that increase oligonucleotide specificity and resistance to nucleases. (B) Booster version 3.0 schematic including 100 adenosines in the 5′ end of the 30-nucleotide guide sequence, which targets the 3′ UTR of the mRNA of interest. The first three nucleotides in both 5′ and 3′ ends have been modified by phosphorothioate and 2′-O-methyl. Below are the sequences of the chemically modified SYNGAP1 and non-specific scrambled control boosters version 3.0. (C) Comparison of estimated dissociation constant ($K_D$) means and standard deviations of PABPC between modified (fully modified and 3NT mod) and unmodified poly(A) RNA. No statistical significance in binding affinity was found when comparing modified poly(A) RNAs to unmodified poly(A) RNA by one-way ANOVA. (D) *SYNGAP1* mRNA levels 48 h after transfection with the chemically modified *SYNGAP1*-specific or scrambled control boosters, measured by qRT-PCR. *n* = 3 biological

*(legend continued on next page)*

blot analysis performed using probes specific to the booster guide sequence. As shown in Figure 6E, chemically modified boosters are observable after 1 week following transfection, where unmodified boosters are not present at any appreciable level.

We also investigated the influence of the poly(A) stretch on *SYNGAP1* target engagement. PABPC1 is well known to bind 12 As and protect 25 As,[64–66] thus a poly(A) tail of 100 has theoretically four bound polypeptides. Critically, however, there is no evidence that the number of PABPC1 molecules bound has a quantitative influence on mRNA expression.[66] Chemical synthesis of boosters allowed us to robustly test the influence of poly(A) tail length on mRNA expression. Toward this end, we generated five boosters with poly(A) mimetic sequences of 3, 25, 50, 75, and 100 adenosines (Table S1). SH-SY5Y cells were transfected with these boosters and harvested after 48 h. We measured *SYNGAP1* mRNA levels via qRT-PCR. As shown, we observed that all lengths of poly(A) resulted in a robust and significant increase in SYNGAP1 expression, except the three adenosines only. We do note a general trend toward higher expression as the poly(A) stretch increases; however this effect appears negligible (Figure 6F).

Finally, we examined the durability of the booster V.3.0 in *CTNNB1* heterozygous neurons differentiated from human-derived iPSCs. A chemically modified version of the CTNNB1 booster, featuring a poly(A) tail of 50 adenosines and targeting the *CTNNB1* 3′ UTR, was encapsulated in an LNP formulation and introduced to cultured neurons. Cells were harvested 5 days post transfection for protein extraction. Western blot analysis revealed a significant 1.5-fold increase in B-catenin levels compared to the scrambled control (Figure 6G).

In summary, we assert that these findings strongly indicate that chemically synthesizing short RNA oligos with modified chemistry and a minimal poly(A) tail can significantly boost mRNA expression. This warrants deeper exploration as a potential therapeutic approach for haploinsufficiency disorders.

## DISCUSSION

Haploinsufficiency occurs when a mutation causes gene expression to drop approximately 50% of normal levels and this level is insufficient to maintain normal function. In the central nervous system (CNS), reduced gene product can lead to intellectual impairment and neurological deficits.[67,68] Examples such as *SYNGAP1*, *CTNNB1*, *PURA*, and *MECP2* haploinsufficiencies demonstrate symptoms such as intellectual disability and behavioral issues due to chromosomal deletions or various pathogenic variants (missense, nonsense, frameshift). These disorders often arise sporadically without a family history, stemming from numerous *de novo* mutations.[38,59,69] Addressing each mutation individually is impractical due to patient rarity and mutation diversity. Therapeutic strategies focusing on boosting expression from the functional allele show promise in overcoming these challenges and improving patient outcomes.

The difficulties of addressing haploinsufficiency disorders are exemplified by SYNGAP1 deficiency and CTNNB1 Syndrome. *SYNGAP1* mutations account for approximately 1%–8% of sporadic cases of intellectual disability, with *de novo* mutations also linked to autism spectrum disorder and epilepsy.[59] Over 50 loss-of-function mutations in *SYNGAP1* result in conditions such as mental retardation, autosomal dominant 5 (MRD5), characterized by motor delay, sleep disturbances, and behavioral challenges such as hyperexcitability and aggression.[60,70,71] *SYNGAP1* encodes a neuronal Ras and Rap GTPase-activating protein crucial for synaptic structure and plasticity in excitatory glutamatergic neurons during brain development.[8] Despite its critical role, the presence of multiple protein isoforms with diverse functional domains regulating dendritic development, along with the complexity of *SYNGAP1* mutations, presents significant obstacles for developing effective treatments for SYNGAP1-related disorders.

CTNNB1 syndrome is a monogenetic neurodevelopmental disorder caused by *de novo* heterozygous loss-of-function pathogenic variants in the *CTNNB1* gene, which encodes β-catenin, and it is characterized by insufficient β-catenin and cognitive and motor disabilities, with co-morbid intellectual disability (ID) and autism spectrum disorder (ASD) in a subset of individuals. *CTNNB1* is a high-confidence risk gene for ID and ASD, and β-catenin, Wnt pathway is a convergent target of multiple ID- and ASD-linked genes, underscoring its significant disease relevance. Treatment options are currently lacking.

A promising approach for tackling neurological disorders involves RNA-based therapeutics, which offer advantages over conventional protein-targeted or DNA-based therapies.[72] RNA exhibits greater versatility and potency, with enhanced specificity in binding target RNAs through Watson-Crick base pairing compared to small molecules or antibodies. RNA-based therapeutics are also considered safer for gene therapy as they do not integrate into the genome. Moreover, the production of high-purity therapeutic-grade RNA molecules is faster and less costly than traditional small-molecule drugs or recombinant proteins, as demonstrated by the rapid development and straightforward manufacturing of the SARS-CoV-2 mRNA vaccine.[8,73]

Many RNA-based agents developed for neurological diseases, whether approved or undergoing pre-clinical testing, are antisense

repeats, including two technical repeats per experiment. ****Benjamin, Krieger, and Yekutieli t test, $p < 0.00001$. (E) Detection of SYNGAP1 booster V.1.0 and V.3.0 by northern blot. SH-SY5Y cells were transfected with 40 nM boosters and the cells were washed after 24 h. The medium was changed every 3 days and the cells harvested a week after transfection. Whole-tRNA signal from ethidium bromide staining is used as a loading control. (F) *SYNGAP1* mRNA levels in SH-SY5Y cells 48 h after transfection with SYNGAP1 booster V.3.0 (SB7) with different poly(A) stretch lengths: 3(A) tail, 25, 50, 75, and 100 nucleosides. qRT-PCR analysis shows a robust significant increase in the level of *SYNGAP1* mRNA in the presence of poly(A) tail compared to 3(A) tail. Ordinary one-way ANOVA. (G) Protein levels of β-catenin in *CTNNB1* heterozygote, human iPSC-derived neurons, 5 days after transfection with LNP-packed boosters against *CTNNB1* or a non-specific control. Unpaired t test, $^{**}p < 0.005$.

oligonucleotides (ASOs). ASOs consist of short sequences of ribonucleotides or deoxyribonucleotides that are chemically modified to resist nucleases, enhance target affinity, and reduce immune response. Examples include Nusinersen (Spinraza), developed for spinal muscular atrophy (SMA), and various ASOs designed for Duchenne muscular dystrophy, which function by blocking specific splicing events to promote the production of functional proteins.[74–78]

We have developed an innovative RNA therapeutic designed to increase protein levels from genes that exhibit haploinsufficiency. Our technology, known as booster, functions akin to a poly(A) tail mimetic. This unique therapeutic is distinguished by its design and its potential to treat all patients with specific haploinsufficiency, regardless of mutation.

A mRNA booster comprises a sequence of riboadenosines followed by a 30-mer RNA oligonucleotide. This oligonucleotide acts in an antisense manner to a sequence within the 3′ UTR of the target messenger RNA (mRNA), ensuring precise targeting. This design mimics a crucial aspect of mRNA—its 3′ poly(A)poly(A) tail—which is a common feature in nearly all human mRNAs, typically spanning about 200 nucleotides. The poly(A) tail plays a pivotal role in regulating mRNA translation and stability; its removal via deadenylation triggers translational quiescence and mRNA degradation, thereby influencing protein levels.

Cytoplasmic poly(A)-binding protein 1 (PABPC1) plays a key role in this process, exerting significant post-transcriptional control when recruited to mRNA through exogenous methods.[32,79,80] The booster extends PABPC1's interaction with mRNA beyond the natural poly(A) tail's capabilities, thereby enhancing protein expression.

Our mRNA booster technology shows promising results, successfully increasing levels of various target proteins in human cells and mice. These proteins are implicated in neurological disorders characterized by loss of function—*SYNGAP1*, *CTNNB1*, *PURA*, and *MECP2*. Our study revealed a consistent 2-fold increase in both protein and RNA levels, a finding particularly relevant for therapeutic development. This precise control of gene expression is crucial for treating haploinsufficiency disorders, where both insufficient and excessive gene expression can trigger disease phenotypes in humans and animal models.[49,81–83] Additionally, we can achieve fine-tuned gene expression within physiological ranges by screening the 3′ UTR of target genes to identify optimal ASOs and adjusting the booster sequence accordingly.

Furthermore, we have demonstrated that our technology can be chemically modified for prolonged efficacy. Our development of chemically modified boosters for SYNGAP1 and CTNNB1 demonstrated enhanced durability *in vitro*, marking progress toward a robust therapeutic approach. While these *in vitro* duration effects are promising, their translation to human outcomes requires careful evaluation. This consideration is well illustrated by Spinraza, a chemically modified antisense oligonucleotide that shows effects lasting days in cell culture but persists for months in mice and enables an efficient three-times-yearly dosing schedule in human patients.[84,85] Moving forward, we need to comprehensively explore the chemical landscape to establish a robust therapeutic window for booster technology. Future studies will focus on evaluating whether this technology leads to phenotypic improvements in disease models.

## MATERIALS AND METHODS

### Luciferase booster assay

Firefly luciferase (FLuc) boosters were designed as a short DNA oligonucleotide complementary to the 3′ end of a FLuc mRNA, with or without a tail of 10 riboadenosines fused through iSp9 spacer (Table S1). A-tailing was performed using *E. coli* poly(A) polymerase (NEB, catalog no. [Cat#] M0276L) with 270 (pmol) of oWH6361, 1 mM ATP, and 0.25U/μL of poly(A) polymerase, incubated at 37°C for 10 min.

*FLuc* mRNA was *in vitro* transcribed with the HiScribe T7 ARCA mRNA kit (NEB, Cat# E2060S) using a PCR template amplified with oWH6357 and oWH6358 from pWH232 (Table S2). A-tailing was not performed on the *FLuc* mRNA. *RLuc* was amplified from pWH231 by oWH6359 and oWH6360 (Tables S2 and S3), followed by IVT and A-tailing. The quality of the RNAs was confirmed by electrophoresis on agarose gel. FLuc booster (1.8 pmol) and various amounts of *in vitro*-transcribed *FLuc* mRNA (50 and 8.3 nmol) were annealed and co-transfected with 50 ng of *in vitro*-transcribed *RLuc* mRNA into HEK293 cells, using the Lipofectamine MessengerMAX Reagent (Thermo Fisher Scientific, Cat# LMRNA008). FLuc and RLuc activity were measured 40 h post transfection using the dual luciferase assay (Promega Corporation, E2920). FLuc activity was normalized to RLuc to account for transfection variations.

### Design and generation of poly(A) tail mimetics for endogenous mRNAs

Short RNA molecules complementary to different regions of the 3′ UTR of the gene of the interest were designed as described previously.[86,87] Using Nygenome online tool (http://cas13design.nygenome.org), the predicted sequences with the highest score were selected to be synthesized as mRNA boosters. Our design comprises (1) a short complementary region that hybridizes with the target mRNA; (2) a poly(A) tail that is located either in 3′ or 5′ end of the complementary sequence, or (3) 5′ end and/or 3′ end chemical modifications. In this study, we used different protocols to generate mRNA boosters to improve their design and efficiency (all different versions of booster are listed in Table S1).

Version 1.0 boosters were synthesized as 30-mer RNA oligonucleotides (including two deoxyribonucleotides in 5′ to limit degradation by ribonucleases) obtained from IDT, and A-tailed in 3′ with *E. coli* poly(A) polymerase (NEB cat# M0276). Briefly, reactions with 200 μL total volume were set up with 1 nmol oligonucleotide, 1× poly(A) polymerase buffer, 1.5 mM ATP, and 0.25 U/μL poly(A) polymerase and incubated at 37°C for 1 h. The polyadenylated

oligonucleotides were purified by phenol/chloroform extraction, followed by chloroform extraction, and precipitated with 0.3 M sodium acetate and the same volume of 100% isopropanol. They were washed twice with 750 μL of 75% EtOH, air dried, and resuspended in RNase-free water. To investigate the quality and the size of the polyadenylated oligonucleotides, about 4 pmol was analyzed by denaturing polyacrylamide gel electrophoresis (PAGE: 8 M urea, 6% polyacrylamide 19:1, 1× TBE).

Version 2.0 Boosters (used in Figures 2, 3, and 5) have shorter poly(A) tails and were synthesized by IVT. Short oligos coupled to a tail of 50 adenosines in 5′ (V.2.1) or two poly(A) tails (one in 5′ and one in 3′ of the oligonucleotide, V.2.2) were ordered as single-stranded DNA (ssDNA) from IDT. They were used for DNA assembly in pBluescript II (SK+) containing T7 RNA polymerase promoter (pJC1351) linearized with ECORI? using NEBuilder HiFi DNA Assembly (E5520) and following the manufacturer's protocol. The plasmids containing the inserts were confirmed by Sanger sequencing (Table S3) and digested with SacI restriction enzyme, or used as a template for PCR using universal M13 forward and reverse primers, to create DNA templates for IVT. The RNAs were synthesized with the HiScribe T7 High Yield RNA Synthesis Kit (NEB, USA, Cat# E2040) following the manufacturer's protocol and chemically modified by adding a cap analog to the 5′ end using m7G(5′)ppp(5′)G RNA Cap Structure Analog (10 μmol) (New England BioLabs, Ipswich, MA). Distinct boosters were designed to target different regions on the 3′ UTR of *MECP2*, *SYNGAP1*, *CTNNB1*, and *PURA* genes (Tables S1 and S2).

Version 3.0 Boosters (used in Figure 6) were chemically synthesized by GenScript Biotech (Piscataway, NJ). They are composed of the 30-ribonucleotide guide sequence with a 100 polyriboadenosine tail in 5′. The first three nucleotides in 5′ and the last three nucleotides in the 3′ end are chemically modified with 2′-O-methyl and PS linkage. The oligos were reconstructed in RNase-free water at a final concentration of 100 μM.

### Mammalian cell culture and transfection

We tested our mRNA booster technology in HEK293 (ATCC CRL 1573), HEK293 SuperTopFlash (STF),[88] and SH-SY5Y (ATCC CRL 2266) cell lines as well as iPSC-derived neuronal cells (see below).

HEK293 and HEK293-STF cell lines were grown in Dulbecco's modified Eagle's medium (DMEM) with 10% FBS, and SH-SY5Y cell lines were grown in Eagle's minimum essential medium (EMEM) with 10% FBS following ATCC guidelines. After reaching 70% confluency, cells were transfected with boosters using Lipofectamine Messenger MAX reagent (Thermo Fisher Scientific, CA, USA, product no. LMRNA008) following the manufacturer's protocol. The cells were harvested for further investigation at the times indicated in the Results.

### Neuronal culture and booster transfection

*CTNNB1* heterozygous loss-of-function KOLF2.2J human pluripotent stem cells (iPSCs) (JAX Genomic Medicine Core and JAX Cellular Engineering Service) were generated using CRISPR-Cas9 editing. iPSCs were maintained in mTeSR Plus (Stemcell, Cat# 100-0276) medium until differentiation on Matrigel hESC-Qualified Matrix, LDEV-free (Corning, Cat# 354277)-coated tissue culture dishes.

Differentiation is based on a modified version of a previously described protocol.[89] Briefly, iPSCs were transduced with pTet-O-NGN2-puro (MOI: 8) and Fudelta GW-rtTA (MOI: 3.5) lentivirus (Alstem, custom order) then transferred to tissue culture dishes coated with Matrigel Growth Factor Reduced matrix (Corning, Cat# 354230). At near confluency, cells were changed to KSR medium containing doxycycline (Sigma, Cat# D9891) to induce NGN2 expression then transitioned over the next 2 days to N2B medium containing both doxycycline and puromycin (Thermo Fisher, Cat# A11138-03) to select for cells expressing the lentiviral construct before being frozen back at the day 4 (D4) precursor stage. D4 precursor neurons were then thawed onto tissue culture plates coated with poly-L-ornithine (Sigma, Cat# P4957) and laminin (Sigma, Cat# L2020) in iNeuron medium containing doxycycline, puromycin, B27 supplement (Thermo Fisher, Cat# 17504-044), and the human growth factors BDNF/CNTF/GDNF (Peprotech, Cat# 450-02, 450-13, and 450-10). After a medium change the next day, the cells were allowed to differentiate until day 9, at which point the medium was supplemented with Ara-C (Sigma, Cat# C1768) to inhibit glial proliferation and puromycin/doxycycline were removed. Half medium changes were then done every 4 days until day 17, at which point neurons were transfected with the RNA booster.

The *CTNNB1* RNA booster was packaged in various LNP formulations (Table S4) and added to the neuronal medium. To facilitate transfection of the RNA booster, medium was supplemented with ApoE3/ApoE4 (Peprotech, Cat# 350-02 and 350-04) at 5 μg/mL each.[90] Neurons were then lysed at 48 h for analysis of β-catenin expression.

### RNA analysis

To isolate total RNA from cultured cells, the culture medium was removed and TRIzol Reagent (Thermo Fisher Scientific, Cat# 15596026) was added directly to the cells. Total RNA was extracted using the Direct-zol RNA kit (Zymo Research), following the manufacturer's protocol. cDNA was synthesized using SuperScript II First-Strand Synthesis System (Invitrogen). qPCR was performed using the LightCycler 480 II instrument (Roche Lifescience) and KAPA SYBR FAST qPCR 2× master mix (KAPA Biosystems, 07959621001, KK4651). The data were analyzed using the Cp value and normalized to either *ACTB* or *GAPDH* as a housekeeping gene.

### Protein analysis

Whole-cell proteins were isolated using RIPA buffer (Radioimmunoprecipitation assay buffer) adding 1% Halt Protease and Phosphatase Inhibitor Cocktail (100×) (Thermo Scientific, Cat# 78440). Western blotting was performed using TGX 4%–15% precast gels (Bio-Rad) and transferred to the polyvinylidene fluoride (PVDF) membrane (0.45 μm) using Bio-Rad Transfer-Blot Turbo Transfer System

RTA Transfer kits (Cat# 1704270). The following antibodies were used for immunoblotting according to the manufacturer's suggested concentrations; anti-GAPDH (6C5) (Santa Cruz Biotechnology), anti-MECP2 (D4F3) (Cell Signaling Technology), anti-SYNGAP (Cat# 19739-1-AP) (Proteintech), β-Catenin Antibody (Amino-terminal Antigen) (Cat# 9581Cell Signaling Technology), IRDye 680LT, goat anti-mouse (lot# D20920-15, Li-COR), IRDye 800CW, and goat anti-rabbit (lot# D30322, Li-COR).

### EMSA

EMSA was used to determine the affinity of the RNA-recognition motifs (RRM1-4) of the human poly-adenylate-binding protein cytoplasmic 1 (PABPC1) protein to modified and unmodified poly(A) tails 15 nucleotides in length. PABPC1 RRM1-4 (amino acids 1–370, Uniprot #P11940) contained an N-terminal 6xHis-Sumo tag and was obtained from Cusabio (Cat# CSB-EP017352HU, lot# DA05670a7g0). This protein construct is referred to here as PABPC. The lyophilized PABPC was reconstituted in 1× phosphate-buffered saline (PBS), with 50% glycerol, and stored at −20°C. All RNA probes were synthesized with a Cy5-fluorophore in the 3′ end of the RNA. The 15-nucleotide, unmodified poly(A)-RNA (unmodified) was obtained from IDT. The 15-nucleotide, poly(A) RNAs with 2′-O-methyl and phosphorothioate modifications in the first three nucleotides, or all 15 nucleotides, are referred to as 3NT Mod and fully modified, respectively. These modified RNAs were obtained from Genscript. A 10× binding buffer was created per Kuhn and Pieler,[91] consisting of 1 M NaCl, 50 mM $MgCl_2$, 5 mM EDTA, 0.1% Nonidet-p40, and 1 mg/mL bovine serum albumin.

EMSAs were conducted as previously described[91] with some modification. Briefly, PABPC was serial diluted to create a gradient of PABPC concentrations of 10 μL volume each: 4 μM, 2 μM, 1 μM, 500 nM, 250 nM, 125 nM, 63 nM, 31 nM, and 16 nM (with a no-protein control). A 2× RNA mastermix was created with 20 nM unmodified, 10% glycerol, and 2× binding buffer. 10 μL of 2× mastermix was mixed into PABPC dilutions by pipetting to create the binding reactions. Final concentrations of PABPC ranged from 2 μM to 8 nM, RNA probe at a concentration of 10 nM, and binding buffer was at a final concentration of 1×. A 6% native polyacrylamide gel (with 0.01% Triton X-100) was pre-run without sample for 30 min at 100 V for at least 30 min at 4°C, and wells were cleared of debris by syringe and needle. The binding reactions were incubated in the dark at room temperature for 15 min, loaded onto the gel, and run at 150 V for 25 min at 4°C. Gels were visualized with an Amersham Typhoon (Cytiva), analyzed, and binding curves plotted on R, and bar graph with ANOVA analysis plotted with Prism 9.

EMSAs conducted with 3NT Mod and fully modified were carried out similarly, with two exceptions: (1) the final PABPC gradient ranged from 1,000 to 4 nM, and (2) final RNA probe concentration was 5 nM.

### Mice

All procedures conducted on the mice were approved by the Animal Care and Use Committee of Johns Hopkins University (protocols #MO23E31 and M021E409). The animal care and use programs at Johns Hopkins University meet the requirements of the Federal Law (89-544 and 91-579) and National Institutes of Health (NIH) regulations and are also accredited by the American Association for Accreditation of Laboratory Animal Care (AAALAC). Animals were group housed on a 12-h:12-h light:dark schedule in the Johns Hopkins University Homewood Central Facility (Mudd Hall) and fed with food and water *ad libitum* as appropriate.

For tail vein injection, 6- to 8-week-old female BALB/c mice (Jackson Laboratory) were used. For intracranial injection, adult (24–30 g) CD-1 mice of mixed genders (Charles River Laboratories, USA) were used.

Mice were generally fed a diet containing low fiber (5%), protein (20%), and fat (5%–10%). The pelleted feed was supplied. Mice were supplied feed free choice, and they ate 4–5 g a day (12 g/100 g body weight/day). Water was supplied free choice and they usually drank 3–5 mL a day (1.5 mL/10 g body weight/day). Water was supplied using automatic waterers. Mouse rooms were maintained at 30%–70% relative humidity and a temperature of 18°C–26°C (64°F–79°F) with at least 10 room air changes per hour. The mice were housed in standard shoebox cages with filter tops. Mice were provided with corncob as bedding.

### *In vivo* delivery and analysis

#### *LNP synthesis and characterization*

DLin-MC3-DMA was purchased from MedKoo Biosciences. DOTAP, DDAB, DSPC, DOPE, 18PG (sodium salt), and 14PA were purchased from Avanti Polar Lipids. Cholesterol was purchased from Sigma. DMG-PEG (MW 2000) (DMG-PEG2000) was purchased from NOF America Corporation (Figure S4; Table S4).

An organic phase was prepared by dissolving a mixture of the helper lipids (DOTAP, DDAB, DOPE, DSPC, 14PA, or 18PG), cholesterol, DMG-PEG2000, and Dlin-MC3 DMA in ethanol at a predetermined molar ratio. The aqueous phase was composed of synthesized RNA dissolved in 25 mM magnesium acetate buffer (pH 4.0, Fisher). All RNA samples were stored at −80°C and thawed on ice prior to use.

For large-scale LNP production, the ethanol and aqueous phases were combined at a 3:1 ratio using syringe pumps in an (Flash NanoComplexation) FNC device, following previously established methods.[46,92,93] The resulting LNPs were dialyzed against PBS using a 100,000 MWCO cassette (Fisher) at 4°C for 24 h and subsequently stored at 4°C until injection.

The size, polydispersity index, and zeta potentials of the LNPs were determined using dynamic light scattering (ZetaPALS, Brookhaven Instruments), with diameters reported as the intensity mean average.

#### *Tail vein injection*

For intravenous (i.v.) injection study, the LNPs were injected i.v. via mouse lateral tail vein at a predetermined dose per mouse. For animal

welfare monitoring, after dosing, the animals were monitored at 24 and 48 h to ensure that they were not harmed. For animal euthanasia, mice were euthanized by $CO_2$ asphyxiation. The death of the animal was verified by cervical dislocation.

#### *Intracranial injection*

Deep brain injection was performed using a controlled stereotaxic injection method as described previously and was modified to target the brain region of interest.[94,95] All metal tools in direct contact with the animal subjects were either autoclaved or bead-sterilized (Fine Science Tools) before use, and all plastic tools in direct contact with the animal subjects were disinfected with 70% ethanol and rinsed with sterile deionized (DI) water and 1 × PBS before use.

Mice were anesthetized by isoflurane. The degree of anesthesia was verified via toe pinch before surgery. A homeothermic blanket (Harvard Apparatus) was set to 37°C and placed underneath the anesthetized mouse. The anesthetized mouse was placed in a stereotaxic frame (Stoelting) equipped with two ear bars and one nose clamp. Puralube vet ointment (Dechra Pharmaceuticals) was applied to moisturize eyes throughout the operation. Hair-removal lotion (Nair, Church & Dwight) was applied to the scalp for depilation and Betadine surgical scrub (Purdue Products) was applied to sterilize the depilated scalp skin. The shaved and disinfected scalp was incised to expose a ca. 6 × 8-mm area of the skull.

A 1-mm-diameter burr hole was made with a dental drill (Micromotor with On/Off Pedal 110/220, Grobet USA) at stereotaxic coordinates: anteroposterior, ca. −2 mm; mediolateral, ca. 1 mm. A sterilized 0-80 set screw (McMaster-Carr) was inserted into the 3D Printed Head Fixation Bracket and the burr hole to a depth of 800 μm, secured with Metabond adhesive cement (Parkell). A second 1-mm-diameter burr hole was made for deep brain nanoparticle infusion into the hippocampus at the following coordinates: anteroposterior, ca. 1.5 mm; mediolateral, ca. 4 mm. The dura was incised and resected with a sterile 27-gauge needle (PrecisionGlide, Becton Dickinson), and sterile 1× PBS was used to keep the skull moist during surgery.

Nanoparticles were injected into the hippocampus along the septotemporal axis using a controlled injection method. A nickel-titanium superelastic tube (internal diameter [ID] 220 μm, outside diameter [OD] 260 μm; Piertech) was molded to the shape of the customized trajectory via 500°C annealing to create the desired curvature matching the mouse hippocampus. Sterilized superelastic tubing was inserted into a micropipette holder (1-HL-U, Molecular Devices) fixed to the displacement platform. The micropipette holder was attached to an injector as described previously, which was mounted on a syringe pump (PHD 2000, Harvard Apparatus).

The syringe containing the memory alloy was inserted into the brain tissue through a previously drilled hole to the target coordinates. Controlled injections were performed by synchronizing the syringe pump with the displacement stage at a nanoparticle injection rate of 5 mL $h^{-1}$ and a displacement stage retraction rate of 0.2–0.5 mm $s^{-1}$. The total injection volume was ca. 20 μL. After implantation, the scalp was closed with 3M Vetbond tissue adhesive.

After surgery, each mouse was returned to a cage placed on a 37°C heating pad. The activity of the mouse was monitored regularly until it was fully recovered from anesthesia. Buprenex (Buprenorphine, Patterson Veterinary Supply) analgesia was given intraperitoneally at a dose of 0.05 mg $kg^{-1}$ body weight every 12 h.

For animal euthanasia, mice were euthanized by $CO_2$ asphyxiation. The death of the animal was verified by cervical dislocation.

#### *Analysis of booster efficacy* in vivo

Brains were harvested and dissected so that different brain regions were used for RNA and protein extraction. To extract RNA, Trizol was added to the frozen tissue and homogenized, and RNA was isolated using Direct-zol RNA miniprep kit (Zymo Research). To extract protein, RIPA buffer + 1× protease/phosphatase inhibitor was added to frozen tissue and, after homogenizing, cell pellet and genomic DNA were precipitated by centrifuging at 14,000 rpm for 10 min. The collected supernatants were used for SDS-PAGE.

#### Statistical analysis

A two-tailed t test or a one-way, two-way ANOVA was performed when comparing two groups or more than two groups. Assumptions of equal variances and normality were tested to assess *post hoc* analysis (Student's, Welch's, or Mann-Whitney's t test). Statistical analysis was performed using Prism 9.0 (GraphPad) and Microsoft Excel (16.61.1). Whenever applicable, mean and standard deviation are reported (or median if not normally distributed). A difference is considered significant when $p < 0.05$ (*$p < 0.05$, **$p < 0.001$, ***$p < 0.001$, ****$p < 0.0001$).

## DATA AND CODE AVAILABILITY

The authors confirm that the data supporting the findings of this study are available within the article and its supplementary materials.

## ACKNOWLEDGMENTS

This work was supported by a charitable gift from Mr. Carl Hull and Mrs. Nanci Hull (to J.C.). Further support was provided by the SynGAP Research Fund (J.C.), Bisciotti Translational Fund (J.C.), Maryland Innovation Initiative Award (J.C.), and the National Institutes of Health: R35GM144114 (J.C.), R21NS131841 (M.H.J.), U01AI155313 (HQM), and CTNNB1 Connect and Cure, Inc (M.H.J.). The authors thank the members of the Coller lab for discussion and comments. We also thank Austin Sponaugle, for assistant with figures. In addition, we thank Drs. Sashank Reddy, Shalini Oberdoerffer, Rick Huganir, and Annie Vemu for helpful discussions during the development of this work.

## AUTHOR CONTRIBUTIONS

B.T., S.M., and J.C. conceptualized the study and wrote the manuscript. B.T., Y.Z., C.L., J.M.A., J.M. Y.S., and K.R.M. conducted experiments and data collection. B.T., S.M., Y.Z., C.L., J.M.A., and K.R.M. selected the methodology to be used in this study under supervision of J.C., M.H.J., W.H., D.L., and H.-Q.M. Data visualized and analyzed by B.T. under supervision of J.C. J.C. directed the project and provided resources and funding for the study.

## DECLARATION OF INTERESTS

A patent application based on this work has been submitted to the USPTO.

## SUPPLEMENTAL INFORMATION

Supplemental information can be found online at https://doi.org/10.1016/j.omtn.2025.102453.

## REFERENCES


1. Veitia, R.A., and Potier, M.C. (2015). Gene dosage imbalances: action, reaction, and models. Trends Biochem. Sci. *40*, 309–317.
2. Veitia, R.A. (2002). Exploring the etiology of haploinsufficiency. Bioessays. *24*, 175–184.
3. Johnson, A.F., Nguyen, H.T., and Veitia, R.A. (2019). Causes and effects of haploinsufficiency. Biol. Rev. *94*, 1774–1785.
4. Escayg, A., MacDonald, B.T., Meisler, M.H., Baulac, S., Huberfeld, G., An-Gourfinkel, I., Brice, A., LeGuern, E., Moulard, B., Chaigne, D., et al. (2000). Mutations of SCN1A, encoding a neuronal sodium channel, in two families with GEFS+ 2. Nat. Genet. *24*, 343–345.
5. Zhu, Y., Zhu, L., Wang, X., and Jin, H. (2022). RNA-based therapeutics: an overview and prospectus. Cell Death Dis. *13*, 644.
6. Bajan, S., and Hutvagner, G. (2020). RNA-based therapeutics: from antisense oligonucleotides to miRNAs. Cells *9*, 137.
7. Leppek, K., Byeon, G.W., Kladwang, W., Wayment-Steele, H.K., Kerr, C.H., Xu, A.F., Kim, D.S., Topkar, V.V., Choe, C., Rothschild, D., et al. (2022). Combinatorial optimization of mRNA structure, stability, and translation for RNA-based therapeutics. Nat. Commun. *13*, 1536.
8. Lee, M.-J., Lee, I., and Wang, K. (2022). Recent Advances in RNA Therapy and Its Carriers to Treat the Single-Gene Neurological Disorders. Biomedicines *10*, 158.
9. Qin, S., Tang, X., Chen, Y., Chen, K., Fan, N., Xiao, W., Zheng, Q., Li, G., Teng, Y., Wu, M., and Song, X. (2022). mRNA-based therapeutics: powerful and versatile tools to combat diseases. Signal Transduct. Target. Ther. *7*, 166.
10. Holstege, F.C., Jennings, E.G., Wyrick, J.J., Lee, T.I., Hengartner, C.J., Green, M.R., Golub, T.R., Lander, E.S., and Young, R.A. (1998). Dissecting the regulatory circuitry of a eukaryotic genome. Cell *95*, 717–728.
11. Eisen, T.J., Eichhorn, S.W., Subtelny, A.O., Lin, K.S., McGeary, S.E., Gupta, S., and Bartel, D.P. (2020). The dynamics of cytoplasmic mRNA metabolism. Mol. Cell *77*, 786–799.e10.
12. Passmore, L.A., and Coller, J. (2022). Roles of mRNA poly (A) tails in regulation of eukaryotic gene expression. Nat. Rev. Mol. Cell Biol. *23*, 93–106.
13. Gallie, D.R. (1991). The cap and poly (A) tail function synergistically to regulate mRNA translational efficiency. Genes Dev. *5*, 2108–2116.
14. Khan, Z., Ford, M.J., Cusanovich, D.A., Mitrano, A., Pritchard, J.K., and Gilad, Y. (2013). Primate transcript and protein expression levels evolve under compensatory selection pressures. Science *342*, 1100–1104.
15. Wang, Z.-Y., Leushkin, E., Liechti, A., Ovchinnikova, S., Mößinger, K., Brüning, T., Rummel, C., Grützner, F., Cardoso-Moreira, M., Janich, P., et al. (2020). Transcriptome and translatome co-evolution in mammals. Nature *588*, 642–647.
16. Jacobson, A., and Favreau, M. (1983). Possible Involvement of poly (A) in protein synthesks. Nucleic Acids Res. *11*, 6353–6368.
17. Edmonds, M., Vaughan, M.H., Jr., and Nakazato, H. (1971). Polyadenylic acid sequences in the heterogeneous nuclear RNA and rapidly-labeled polyribosomal RNA of HeLa cells: possible evidence for a precursor relationship. Proc. Natl. Acad. Sci. USA *68*, 1336–1340.
18. McLaughlin, C.S., Warner, J.R., Edmonds, M., Nakazato, H., and Vaughan, M.H. (1973). Polyadenylic acid sequences in yeast messenger ribonucleic acid. J. Biol. Chem. *248*, 1466–1471.
19. Baer, B.W., and Kornberg, R.D. (1983). The protein responsible for the repeating structure of cytoplasmic poly (A)-ribonucleoprotein. J. Cell Biol. *96*, 717–721.
20. Schäfer, I.B., Yamashita, M., Schuller, J.M., Schüssler, S., Reichelt, P., Strauss, M., and Conti, E. (2019). Molecular basis for poly (A) RNP architecture and recognition by the Pan2-Pan3 deadenylase. Cell *177*, 1619–1631e1621.
21. Legnini, I., Alles, J., Karaiskos, N., Ayoub, S., and Rajewsky, N. (2019). Full-length mRNA sequencing reveals principles of poly (A) tail length control. bioRxiv, 547034. https://doi.org/10.1101/547034.
22. Preiss, T., and Hentze, M.W. (1998). Dual function of the messenger RNA cap structure in poly (A)-tail-promoted translation in yeast. Nature *392*, 516–520.
23. Kahvejian, A., Svitkin, Y.V., Sukarieh, R., M'Boutchou, M.-N., and Sonenberg, N. (2005). Mammalian poly (A)-binding protein is a eukaryotic translation initiation factor, which acts via multiple mechanisms. Genes Dev. *19*, 104–113.
24. Jackson, R.J., Hellen, C.U.T., and Pestova, T.V. (2010). The mechanism of eukaryotic translation initiation and principles of its regulation. Nat. Rev. Mol. Cell Biol. *11*, 113–127.
25. Wilson, T., and Treisman, R. (1988). Removal of poly (A) and consequent degradation of c-fos mRNA facilitated by 3′ AU-rich sequences. Nature *336*, 396–399.
26. Decker, C.J., and Parker, R. (1993). A turnover pathway for both stable and unstable mRNAs in yeast: evidence for a requirement for deadenylation. Genes Dev. *7*, 1632–1643.
27. Schiavi, S.C., Wellington, C.L., Shyu, A.-B., Chen, C.Y., Greenberg, M.E., and Belasco, J.G. (1994). Multiple elements in the c-fos protein-coding region facilitate mRNA deadenylation and decay by a mechanism coupled to translation. J. Biol. Chem. *269*, 3441–3448.
28. Eckmann, C.R., Rammelt, C., and Wahle, E. (2011). Control of poly (A) tail length. Wiley Interdiscip. Rev. RNA *2*, 348–361.
29. Shao, J., Li, S., Qiu, X., Jiang, J., Zhang, L., Wang, P., Si, Y., Wu, Y., He, M., Xiong, Q., et al. (2024). Engineered poly (A)-surrogates for translational regulation and therapeutic biocomputation in mammalian cells. Cell Res. *34*, 31–46.
30. Chen, H., Liu, D., Guo, J., Aditham, A., Zhou, Y., Tian, J., Luo, S., Ren, J., Hsu, A., Huang, J., et al. (2024). Branched chemically modified poly (A) tails enhance the translation capacity of mRNA. Nat. Biotechnol. 1–10.
31. Cao, J., and Kuyumcu-Martinez, M.N. (2023). Alternative polyadenylation regulation in cardiac development and cardiovascular disease. Cardiovasc. Res. *119*, 1324–1335.
32. Coller, J.M., Gray, N.K., and Wickens, M.P. (1998). mRNA stabilization by poly(A) binding protein is independent of poly(A) and requires translation. Genes Dev. *12*, 3226–3235. https://doi.org/10.1101/gad.12.20.3226.
33. Sheets, M.D., Wu, M., and Wickens, M. (1995). Polyadenylation of c-mos mRNA as a control point in Xenopus meiotic maturation. Nature *374*, 511–516.
34. Wessels, H.-H., Méndez-Mancilla, A., Guo, X., Legut, M., Daniloski, Z., and Sanjana, N.E. (2020). Massively parallel Cas13 screens reveal principles for guide RNA design. Nat. Biotechnol. *38*, 722–727.
35. Presnyak, V., Alhusaini, N., Chen, Y.-H., Martin, S., Morris, N., Kline, N., Olson, S., Weinberg, D., Baker, K.E., Graveley, B.R., and Coller, J. (2015). Codon optimality is a major determinant of mRNA stability. Cell *160*, 1111–1124.
36. Forrest, M.E., Pinkard, O., Martin, S., Sweet, T.J., Hanson, G., and Coller, J. (2020). Codon and amino acid content are associated with mRNA stability in mammalian cells. PLoS One *15*, e0228730.
37. Hamdan, F.F., Srour, M., Capo-Chichi, J.-M., Daoud, H., Nassif, C., Patry, L., Massicotte, C., Ambalavanan, A., Spiegelman, D., Diallo, O., et al. (2014). De novo mutations in moderate or severe intellectual disability. PLoS Genet. *10*, e1004772.
38. Parker, M.J., Fryer, A.E., Shears, D.J., Lachlan, K.L., McKee, S.A., Magee, A.C., Mohammed, S., Vasudevan, P.C., Park, S.M., Benoit, V., et al. (2015). De novo, heterozygous, loss-of-function mutations in SYNGAP1 cause a syndromic form of intellectual disability. Am. J. Med. Genet. A *167A*, 2231–2237.
39. Iossifov, I., O'roak, B.J., Sanders, S.J., Ronemus, M., Krumm, N., Levy, D., Stessman, H.A., Witherspoon, K.T., Vives, L., Patterson, K.E., et al. (2014). The contribution of de novo coding mutations to autism spectrum disorder. Nature *515*, 216–221.
40. O'Roak, B.J., Stessman, H.A., Boyle, E.A., Witherspoon, K.T., Martin, B., Lee, C., Vives, L., Baker, C., Hiatt, J.B., Nickerson, D.A., et al. (2014). Recurrent de novo mutations implicate novel genes underlying simplex autism risk. Nat. Commun. *5*, 5595.
41. (2015). Large-scale discovery of novel genetic causes of developmental disorders. Nature *519*, 223–228.
42. Wilfert, A.B., Sulovari, A., Turner, T.N., Coe, B.P., and Eichler, E.E. (2017). Recurrent de novo mutations in neurodevelopmental disorders: properties and clinical implications. Genome Med. *9*, 101.
43. Ip, J.P.K., Mellios, N., and Sur, M. (2018). Rett syndrome: insights into genetic, molecular and circuit mechanisms. Nat. Rev. Neurosci. *19*, 368–382.

44. Chahrour, M., Jung, S.Y., Shaw, C., Zhou, X., Wong, S.T.C., Qin, J., and Zoghbi, H.Y. (2008). MeCP, a key contributor to neurological disease, activates and represses transcription. Science *320*, 1224–1229. https://doi.org/10.1126/science.1153252.

45. Lu, H., Ash, R.T., He, L., Kee, S.E., Wang, W., Yu, D., Hao, S., Meng, X., Ure, K., Ito-Ishida, A., et al. (2016). Loss and gain of MeCP2 cause similar hippocampal circuit dysfunction that is rescued by deep brain stimulation in a Rett syndrome mouse model. Neuron *91*, 739–747.

46. Zhu, Y., Shen, R., Vuong, I., Reynolds, R.A., Shears, M.J., Yao, Z.C., Hu, Y., Cho, W.J., Kong, J., and Reddy, S.K. (2022). Multi-step screening of DNA/lipid nanoparticles and co-delivery with siRNA to enhance and prolong gene expression. Nat. Commun. *13*, 4282. https://doi.org/10.1038/s41467-022-31993-y.

47. Vashi, N., and Justice, M.J. (2019). Treating Rett syndrome: from mouse models to human therapies. Mamm. Genome *30*, 90–110.

48. Zalosnik, M.I., Fabio, M.C., Bertoldi, M.L., Castañares, C.N., and Degano, A.L. (2021). MeCP2 deficiency exacerbates the neuroinflammatory setting and autoreactive response during an autoimmune challenge. Sci. Rep. *11*, 10997.

49. Zhuang, W., Ye, T., Wang, W., Song, W., and Tan, T. (2023). CTNNB1 in neurodevelopmental disorders. Front. Psychiatry *14*, 1143328.

50. Ho, S.K., Tsang, M.H., Lee, M., Cheng, S.S., Luk, H., Lo, I.F., and Chung, B.H. (2022). CTNNB1 neurodevelopmental disorder. In GeneReviews® [Internet], M.P. Adam, J. Feldman, and G.M. Mirzaa, et al., eds. (Seattle, WA: University of Washington, Seattle), https://www.ncbi.nlm.nih.gov/books/NBK580527/.

51. Sudnawa, K.K., Garber, A., Cohen, R., Calamia, S., Kanner, C.H., Montes, J., Bain, J.M., Fee, R.J., and Chung, W.K. (2024). Clinical phenotypic spectrum of CTNNB1 neurodevelopmental disorder. Clin. Genet. *105*, 523–532.

52. Liu, J., Xiao, Q., Xiao, J., Niu, C., Li, Y., Zhang, X., Zhou, Z., Shu, G., and Yin, G. (2022). Wnt/β-catenin signalling: function, biological mechanisms, and therapeutic opportunities. Signal Transduct. Target. Ther. *7*, 3.

53. Mao, B., Wu, W., Davidson, G., Marhold, J., Li, M., Mechler, B.M., Delius, H., Hoppe, D., Stannek, P., Walter, C., et al. (2002). Kremen proteins are Dickkopf receptors that regulate Wnt/β-catenin signalling. Nature *417*, 664–667.

54. Alves dos Santos, M.T.M., and Smidt, M.P. (2011). En1 and Wnt signaling in midbrain dopaminergic neuronal development. Neural Dev. *6*, 23.

55. Nusse, R., and Clevers, H. (2017). Wnt/β-catenin signaling, disease, and emerging therapeutic modalities. Cell *169*, 985–999.

56. Hamdan, F.F., Daoud, H., Piton, A., Gauthier, J., Dobrzeniecka, S., Krebs, M.-O., Joober, R., Lacaille, J.-C., Nadeau, A., Milunsky, J.M., et al. (2011). De novo SYNGAP1 mutations in nonsyndromic intellectual disability and autism. Biol. Psychiatry *69*, 898–901.

57. Gamache, T.R., Araki, Y., and Huganir, R.L. (2020). Twenty years of SynGAP research: from synapses to cognition. J. Neurosci. *40*, 1596–1605.

58. Berryer, M.H., Hamdan, F.F., Klitten, L.L., Møller, R.S., Carmant, L., Schwartzentruber, J., Patry, L., Dobrzeniecka, S., Rochefort, D., Neugnot-Cerioli, M., et al. (2013). Mutations in SYNGAP1 cause intellectual disability, autism, and a specific form of epilepsy by inducing haploinsufficiency. Hum. Mutat. *34*, 385–394.

59. Agarwal, M., Johnston, M.V., and Stafstrom, C.E. (2019). SYNGAP1 mutations: Clinical, genetic, and pathophysiological features. Int. J. Dev. Neurosci. *78*, 65–76. https://doi.org/10.1016/j.ijdevneu.2019.08.003.

60. Kim, J.H., Liao, D., Lau, L.-F., and Huganir, R.L. (1998). SynGAP: a synaptic RasGAP that associates with the PSD-95/SAP90 protein family. Neuron *20*, 683–691.

61. Porter, K., Komiyama, N.H., Vitalis, T., Kind, P.C., and Grant, S.G.N. (2005). Differential expression of two NMDA receptor interacting proteins, PSD-95 and SynGAP during mouse development. Eur. J. Neurosci. *21*, 351–362.

62. Tucker, M., Valencia-Sanchez, M.A., Staples, R.R., Chen, J., Denis, C.L., and Parker, R. (2001). The transcription factor associated Ccr4 and Caf1 proteins are components of the major cytoplasmic mRNA deadenylase in Saccharomyces cerevisiae. Cell *104*, 377–386.

63. Chen, Y., Khazina, E., Izaurralde, E., and Weichenrieder, O. (2021). Crystal structure and functional properties of the human CCR4-CAF1 deadenylase complex. Nucleic Acids Res. *49*, 6489–6510.

64. Smith, B.L., Gallie, D.R., Le, H., and Hansma, P.K. (1997). Visualization of poly (A)-binding protein complex formation with poly (A) RNA using atomic force microscopy. J. Struct. Biol. *119*, 109–117.

65. Deo, R.C., Bonanno, J.B., Sonenberg, N., and Burley, S.K. (1999). Recognition of polyadenylate RNA by the poly (A)-binding protein. Cell *98*, 835–845.

66. Nicholson-Shaw, A.L., Kofman, E.R., Yeo, G.W., and Pasquinelli, A.E. (2022). Nuclear and cytoplasmic poly (A) binding proteins (PABPs) favor distinct transcripts and isoforms. Nucleic Acids Res. *50*, 4685–4702.

67. Roque, S., Cerciat, M., Gaugué, I., Mora, L., Floch, A.G., de Zamaroczy, M., Heurgué-Hamard, V., and Kervestin, S. (2015). Interaction between the poly (A)-binding protein Pab1 and the eukaryotic release factor eRF3 regulates translation termination but not mRNA decay in Saccharomyces cerevisiae. Rna *21*, 124–134.

68. Kong, Y., Zhou, W., and Sun, Z. (2020). Nuclear receptor corepressors in intellectual disability and autism. Mol. Psychiatry *25*, 2220–2236. https://doi.org/10.1038/s41380-020-0667-y.

69. Carvill, G.L., Matheny, T., Hesselberth, J., and Demarest, S. (2021). Haploinsufficiency, dominant negative, and gain-of-function mechanisms in epilepsy: Matching therapeutic approach to the pathophysiology. Neurotherapeutics *18*, 1500–1514.

70. Chen, H.-J., Rojas-Soto, M., Oguni, A., and Kennedy, M.B. (1998). A synaptic Ras-GTPase activating protein (p135 SynGAP) inhibited by CaM kinase II. Neuron *20*, 895–904.

71. Gamache, T.R., Araki, Y., and Huganir, R.L. (2020). Twenty Years of SynGAP Research: From Synapses to Cognition. J. Neurosci. *40*, 1596–1605. https://doi.org/10.1523/jneurosci.0420-19.2020.

72. Bettini, E., and Locci, M. (2021). SARS-CoV-2 mRNA vaccines: immunological mechanism and beyond. Vaccines *9*, 147.

73. Scharner, J., Figeac, N., Ellis, J.A., and Zammit, P.S. (2015). Ameliorating pathogenesis by removing an exon containing a missense mutation: a potential exon-skipping therapy for laminopathies. Gene Ther. *22*, 503–515.

74. Han, Z., Chen, C., Christiansen, A., Ji, S., Lin, Q., Anumonwo, C., Liu, C., Leiser, S.C., Meena, n., Aznarez, I., et al. (2020). Antisense oligonucleotides increase Scn1a expression and reduce seizures and SUDEP incidence in a mouse model of Dravet syndrome. Sci. Transl. Med. *12*, eaaz6100.

75. Tang, Q., and Khvorova, A. (2024). RNAi-based drug design: considerations and future directions. Nat. Rev. Drug Discov. *23*, 341–364.

76. Hoy, S.M. (2017). Nusinersen: first global approval. Drugs *77*, 473–479.

77. Gidaro, T., and Servais, L. (2019). Nusinersen treatment of spinal muscular atrophy: current knowledge and existing gaps. Dev. Med. Child Neurol. *61*, 19–24.

78. Neil, E.E., and Bisaccia, E.K. (2019). Nusinersen: a novel antisense oligonucleotide for the treatment of spinal muscular atrophy. J. Pediatr. Pharmacol. Ther. *24*, 194–203.

79. Azizi, H., and Papadopoulou, B. (2020). In Vivo Tethering System to Isolate RNA-Binding Proteins Regulating mRNA Decay in Leishmania. Trypanosomatids. Methods Mol. Biol. *2116*, 325–338.

80. Coller, J., and Wickens, M. (2007). Tethered function assays: an adaptable approach to study RNA regulatory proteins. In Methods in Enzymology (Elsevier), pp. 299–321.

81. Carvill, G.L., Matheny, T., Hesselberth, J., and Demarest, S. (2021). Haploinsufficiency, dominant negative, and gain-of-function mechanisms in epilepsy: matching therapeutic approach to the pathophysiology. Neurotherapeutics *18*, 1500–1514.

82. Araki, Y., Gerber, E.E., Rajkovich, K.E., Hong, I., Johnson, R.C., Lee, H.-K., Kirkwood, A., and Huganir, R.L. (2023). Mouse models of SYNGAP1-related intellectual disability. Proc. Natl. Acad. Sci. USA *120*, e2308891120.

83. Lu, H., Ash, R.T., He, L., Kee, S.E., Wang, W., Yu, D., Hao, S., Meng, X., Ure, K., and Ito-Ishida, A. (2016). Loss and Gain of MeCP2 Cause Similar Hippocampal Circuit Dysfunction that Is Rescued by Deep Brain Stimulation in a Rett Syndrome Mouse Model. Neuron *91*, 739–747. https://doi.org/10.1016/j.neuron.2016.07.018.

84. Hua, Y., Vickers, T.A., Baker, B.F., Bennett, C.F., and Krainer, A.R. (2007). Enhancement of SMN2 exon 7 inclusion by antisense oligonucleotides targeting the exon. PLoS Biol. *5*, e73. https://doi.org/10.1371/journal.pbio.0050073.

85. Hua, Y., Vickers, T.A., Okunola, H.L., Bennett, C.F., and Krainer, A.R. (2008). Antisense masking of an hnRNP A1/A2 intronic splicing silencer corrects SMN2 splicing in transgenic mice. Am. J. Hum. Genet. *82*, 834–848. https://doi.org/10.1016/j.ajhg.2008.01.014.

86. Torkzaban, B., Kawalerski, R., and Coller, J. (2022). Development of a tethered mRNA amplifier to increase protein expression. Biotechnol. J. *17*, 2200214.

87. Torkzaban, B., Sponaugle, A., and Coller, J. (2023). Tethered mRNA Amplifier: A Novel Approach to Increase Protein Expression. In Deadenylation: Methods and Protocols (Springer), pp. 161–172.

88. Xu, Q., Wang, Y., Dabdoub, A., Smallwood, P.M., Williams, J., Woods, C., Kelley, M.W., Jiang, L., Tasman, W., Zhang, K., and Nathans, J. (2004). Vascular development in the retina and inner ear: control by Norrin and Frizzled-4, a high-affinity ligand-receptor pair. Cell *116*, 883–895.

89. Zhang, Y., Pak, C., Han, Y., Ahlenius, H., Zhang, Z., Chanda, S., Marro, S., Patzke, C., Acuna, C., Covy, J., et al. (2013). Rapid single-step induction of functional neurons from human pluripotent stem cells. Neuron *78*, 785–798.

90. Rungta, R.L., Choi, H.B., Lin, P.J., Ko, R.W., Ashby, D., Nair, J., Manoharan, M., Cullis, P.R., and MacVicar, B.A. (2013). Lipid nanoparticle delivery of siRNA to silence neuronal gene expression in the brain. Molecular therapy. Nucleic acids *2*, e136.

91. Kühn, U., and Pieler, T. (1996). XenopusPoly (A) binding protein: functional domains in RNA binding and protein–protein interaction. J. Mol. Biol. *256*, 20–30.

92. Zhu, Y., Cai, S.S., Ma, J., Cheng, L., Wei, C., Aggarwal, A., Toh, W.H., Shin, C., Shen, R., and Kong, J. (2024). Optimization of lipid nanoparticles for gene editing of the liver via intraduodenal delivery. Biomaterials *308*, 122559. https://doi.org/10.1016/j.biomaterials.2024.122559.

93. Zhu, Y., Ma, J., Shen, R., Lin, J., Li, S., Lu, X., Stelzel, J.L., Kong, J., Cheng, L., and Vuong, I. (2024). Screening for lipid nanoparticles that modulate the immune activity of helper T cells towards enhanced antitumour activity. Nat. Biomed. Eng. *8*, 544–560. https://doi.org/10.1038/s41551-023-01131-0.

94. Schuhmann, T.G., Jr., Zhou, T., Hong, G., Lee, J.M., Fu, T.M., Park, H.G., Lieber, C.M., Park, H.-G., and Lieber, C.M. (2018). Syringe-injectable mesh electronics for stable chronic rodent electrophysiology. JoVE, e58003.

95. Hong, G., Fu, T.-M., Zhou, T., Schuhmann, T.G., Huang, J., and Lieber, C.M. (2015). Syringe injectable electronics: precise targeted delivery with quantitative input/output connectivity. Nano Lett. *15*, 6979–6984.

OMTN, Volume *36*

## Supplemental information

# Use of polyadenosine tail mimetics to enhance mRNA expression from genes associated with haploinsufficiency disorders

**Bahareh Torkzaban, Yining Zhu, Christian Lopez, Jonathan M. Alexander, Jingyao Ma, Yongzhi Sun, Katharine R. Maschhoff, Wenqian Hu, Michele H. Jacob, Dingchang Lin, Hai-Quan Mao, Sophie Martin, and Jeff Coller**

## Supplemental material

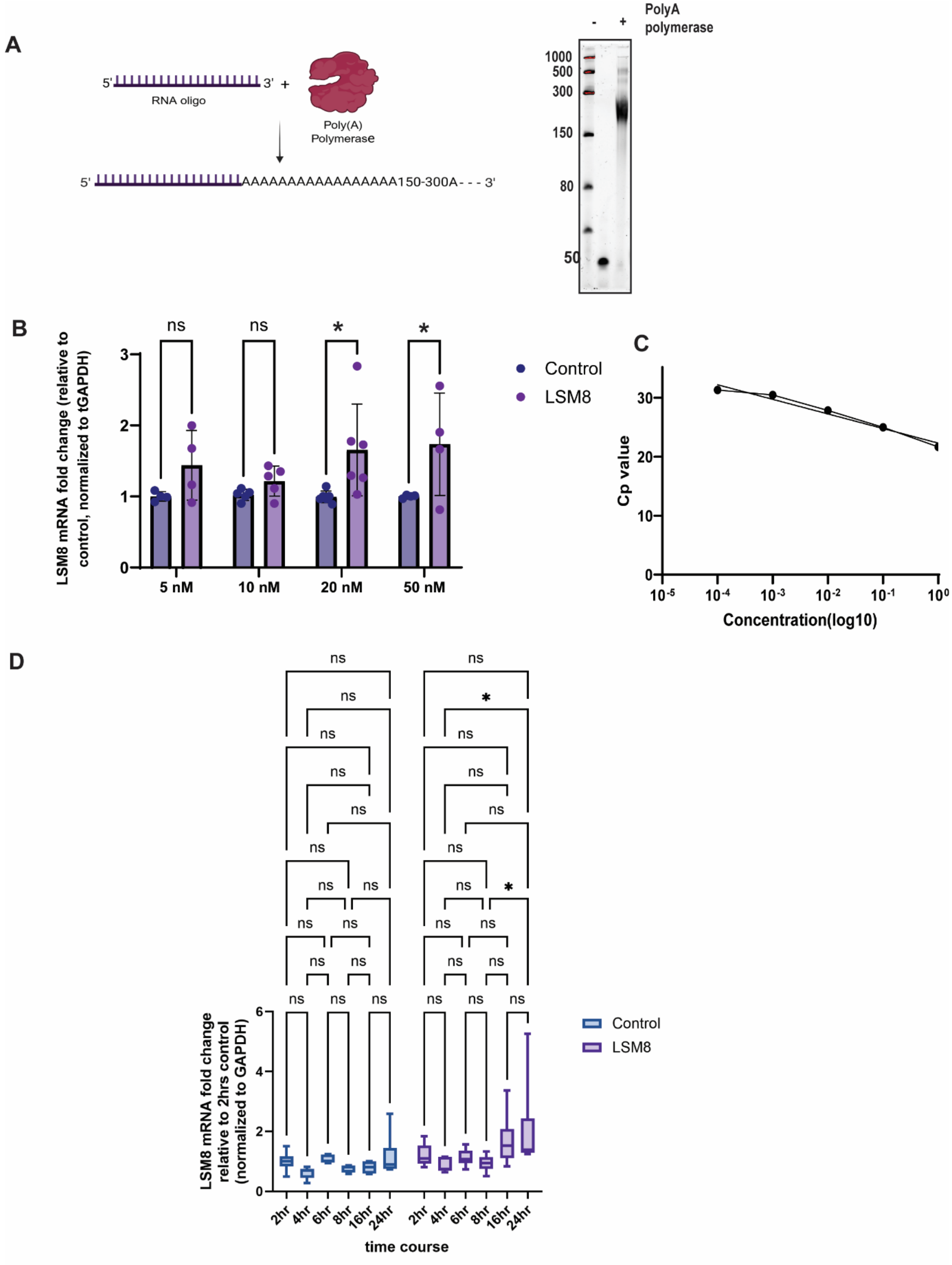

**Figure S1:** (A) Synthesis and quality control of boosters generated using an enzymatic approach that leads to a poly(A) tail of about 150-200 nucleotides; the poly(A) tail was added to the 3'UTR of oligonucleotides with E.Coli poly(A) polymerase, and the polyadenylated oligonucleotides analyzed by polyacrylamide gel electrophoresis. **(B)** HEK293 cells exposed to different doses of LSM8 specific and non-specific booster. 16 hours after transfection, mRNA analysis using RT-qPCR indicates the most effective dose to investigate booster activity for LSM8. **(C)** the graph shows LSM8 primer pair efficiency. **(D)** The box plot indicates no significant time dependent alteration in the level of LSM8 comparing all the control treated time points normalized to 2hr control, while there is significant alteration in the level of LSM8 when normalized to 2hr control between 4 and 24 hrs and 8 and 24 hrs booster treatment. two -way ANOVA,* *p*-value = 0.05.

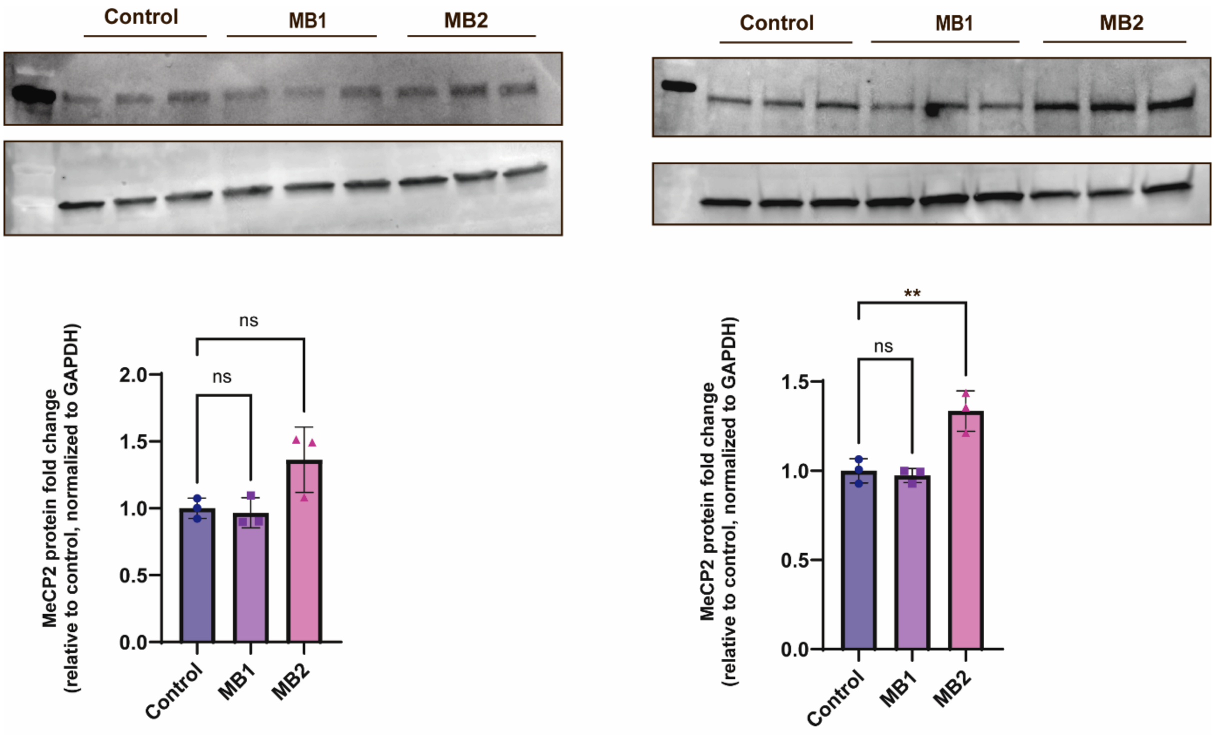


**Figure S2:** Western blotting technical replicates for MeCP2 from liver lysates of 6 weeks-old mice injected with 25ug of LNP-encapsulated boosters (version V2, MB1 and MB2, or non-specific Control) by tail vein injection. The tissues were collected 48 hours after injection. Ordinary one-way ANOVA, * *p*-value = 0.05,** *p*-value = 0.005.

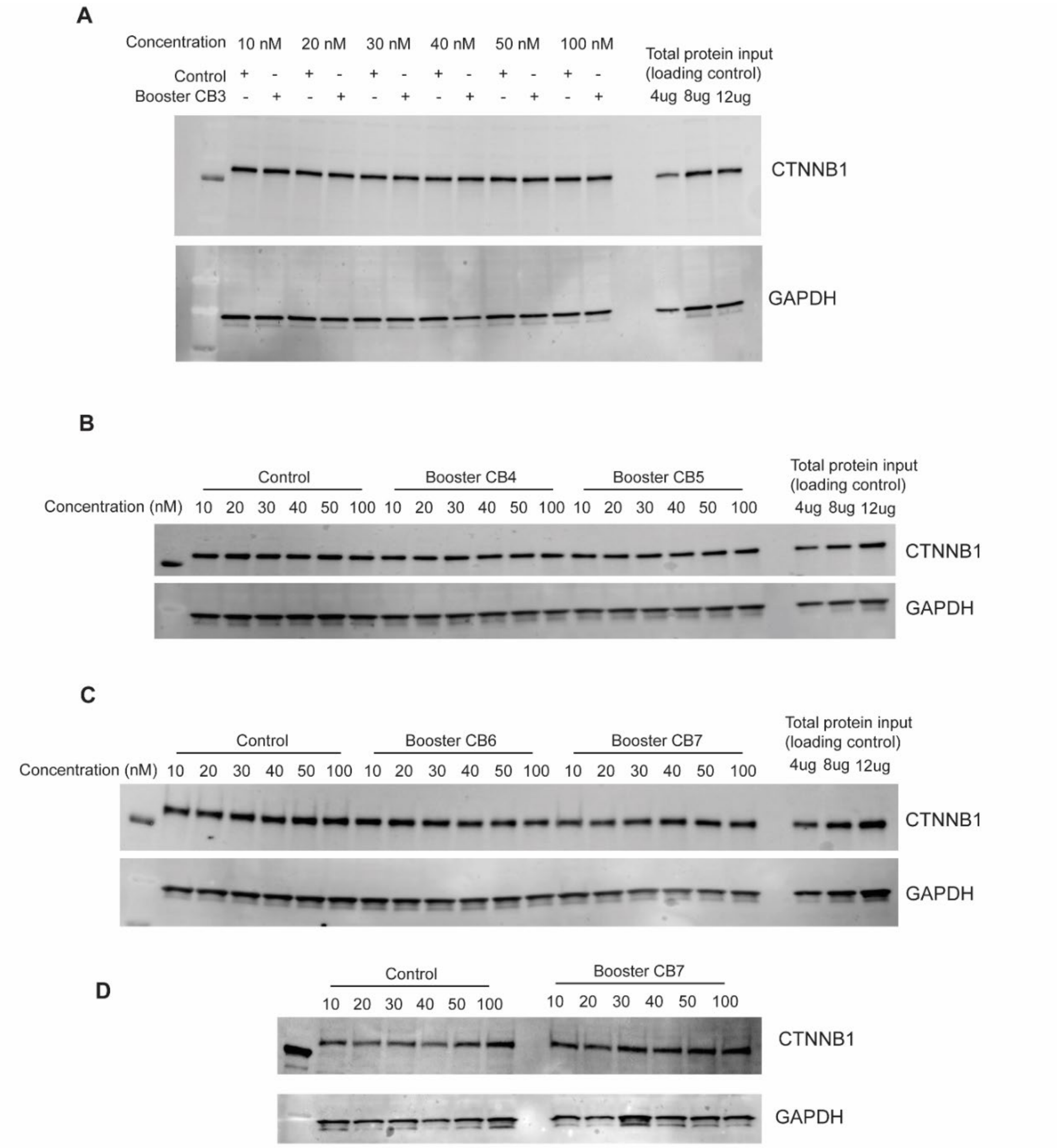


**Figure S3:** The western blot shows CTNNB1 protein level in HEK293 cells. HEK-STF cells treated with different doses of CTNNB1 boosters V.1.0; CB3 **(A)**, CB4 and CB5 **(B)**, CB6 and CB7 **(C)**. Cells were harvested 48 hours after transfection and the protein lysate analyzed by western blotting. Quantitation's for these blots are presented in Figure 4B. **(D)** Additional Western blot representative of CTNNB1 Booster dose response experiment.

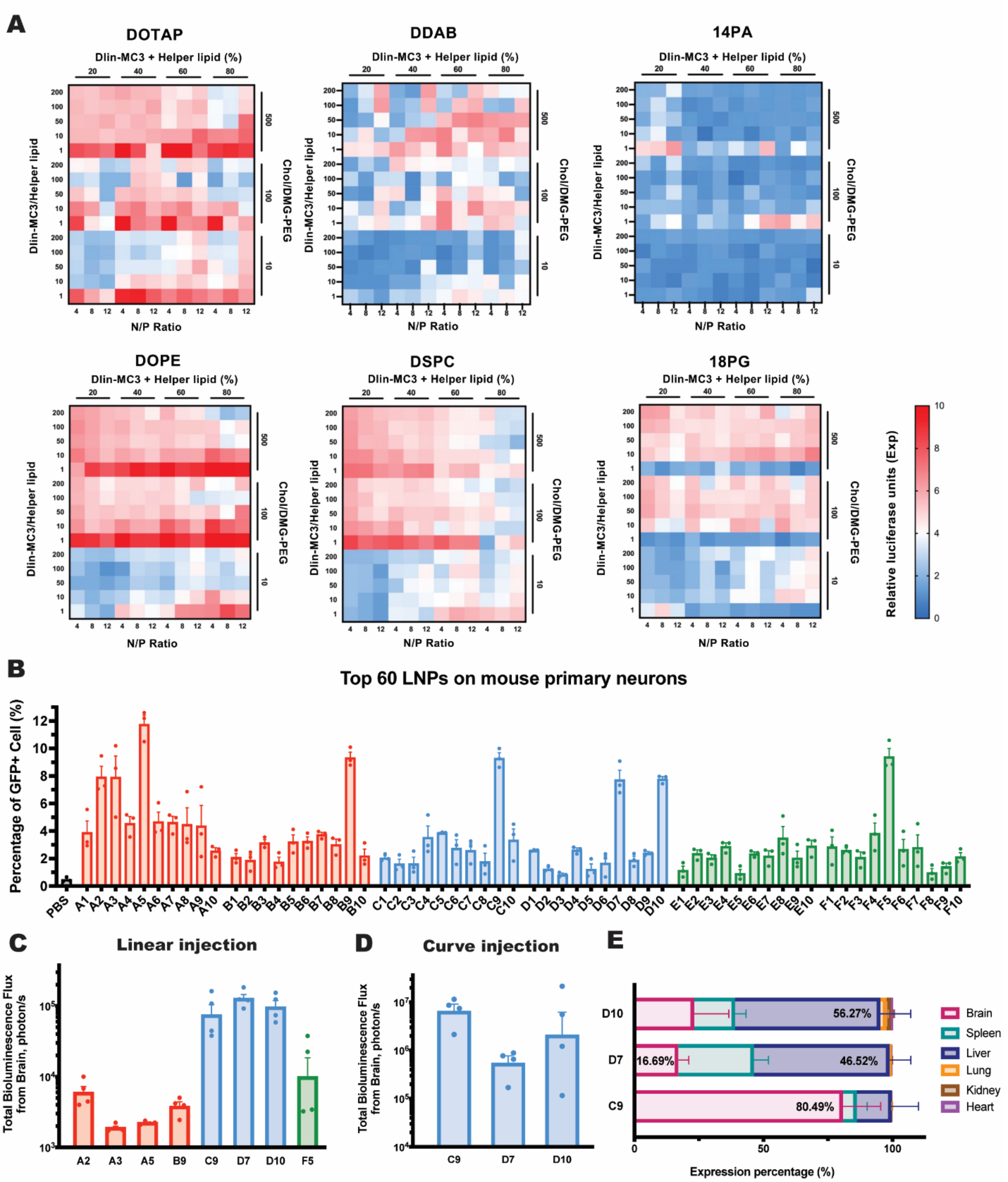


**Figure S4: (A)** Transfection efficiency of pDNA LNPs on Neuro-2a via high-throughput screening platform after 72h incubation. (n= 2) The efficiency of transgene expression of luciferase as a reporter. (pDNA 1μg/well) **(B)** Transfection efficiency of mRNA LNPs on

primary neurons was evaluated via flow cytometry after 24h incubation. (n= 3) The efficiency of transgene expression of GFP as a reporter (mRNA 0.5 μg/well). **(C)** Transfection efficiency of selected mRNA LNPs in the brain via linear injection was evaluated via IVIS after 24h incubation. (n= 4) The efficiency of transgene expression of luciferase as a reporter. (mRNA 5 μg per mouse) **(D-E)** Transfection efficiency of selected mRNA LNPs in the brain via circular injection was evaluated via IVIS after 24h incubation. (n= 4) The efficiency of transgene expression of luciferase as a reporter. (mRNA 5 μg per mouse).

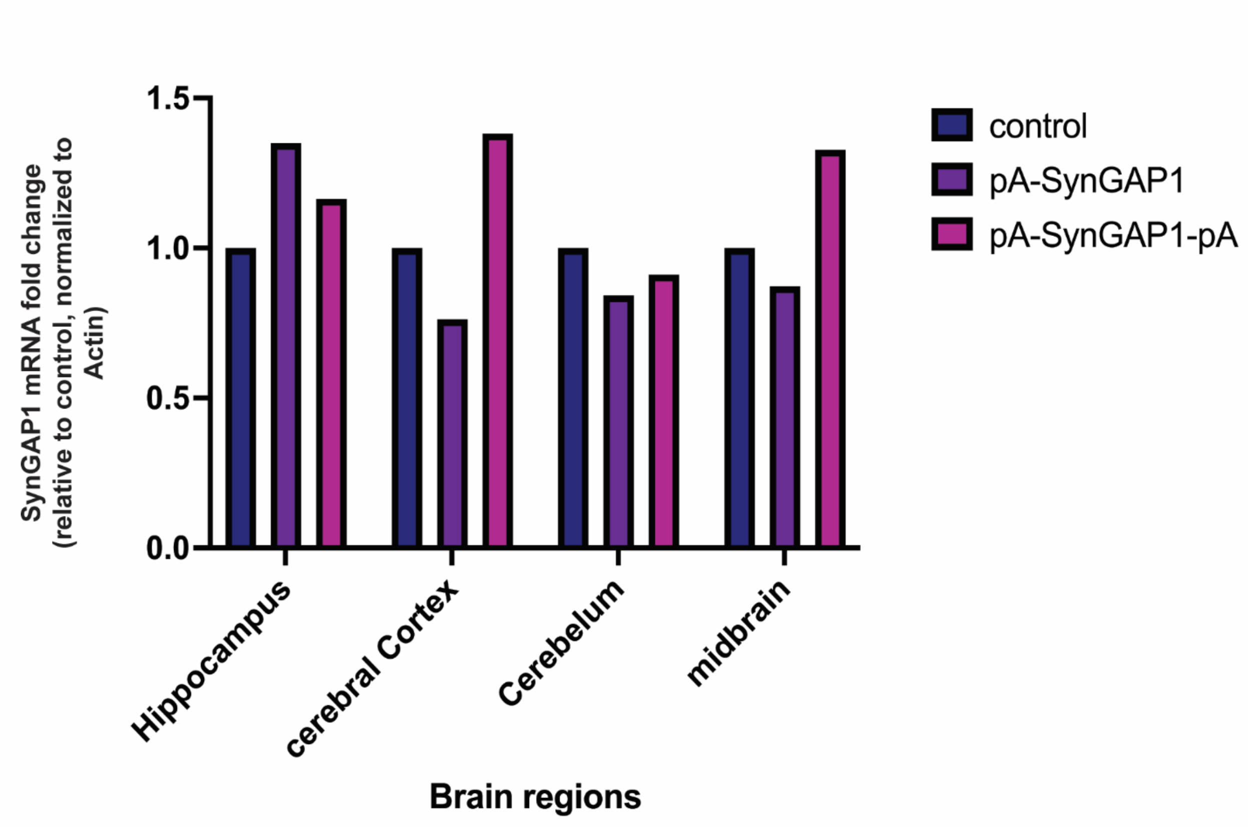


**Figure S5:** mRNA levels of SynGAP1 in the hippocampus of mice injected with 25 ug of LNP-packaged SynGAP1 boosters V.2.0 (SB1 and SB2) or a non-specific scrambled control, measured by RT-qPCR analysis.

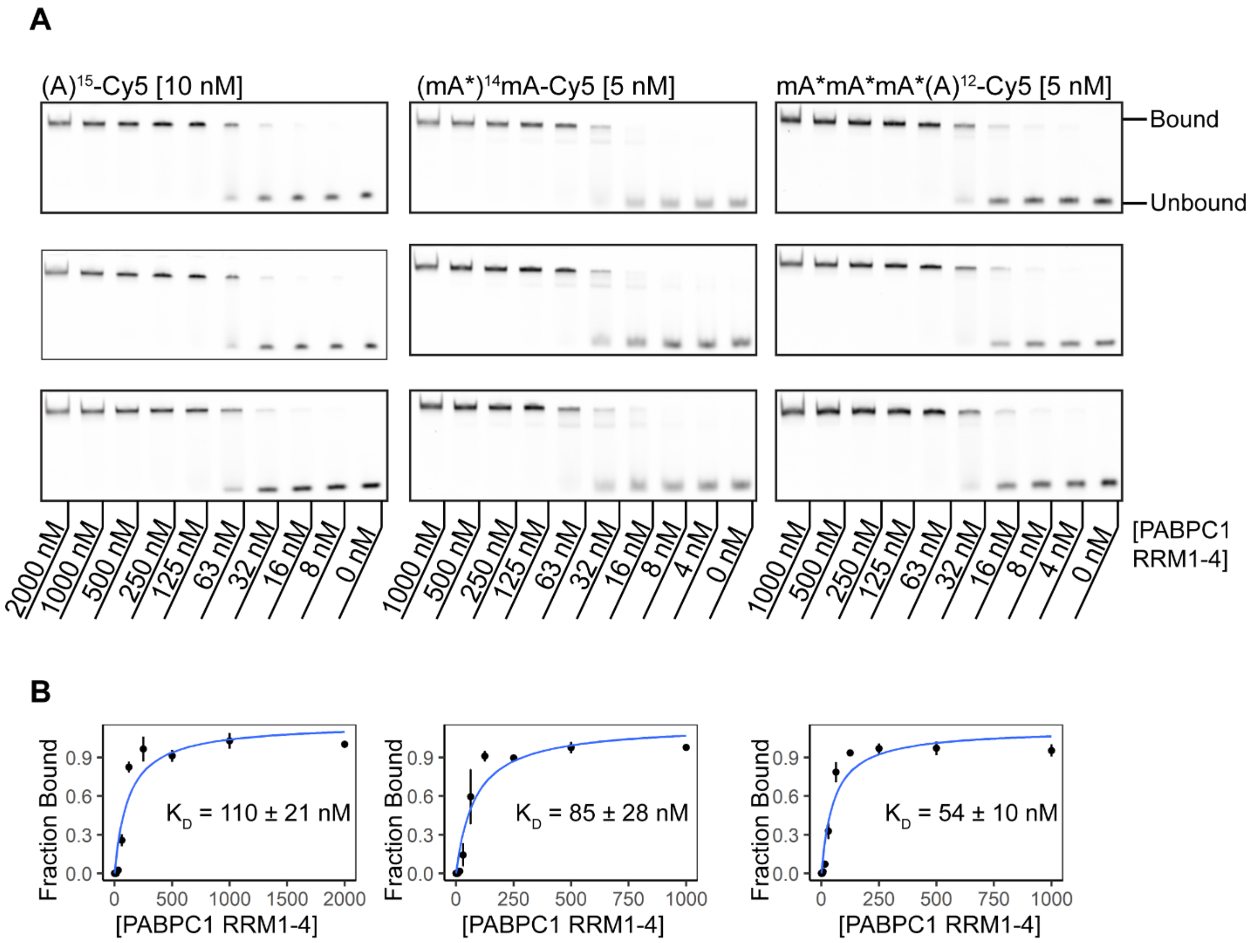


**Figure S6:** Modifications to adenosine bases do not disrupt PABPC binding to poly(A) RNA. **(A)** The binding affinity of the RNA-Recognition Motifs (RRMs) of human PABPC1 (PABPC) to unmodified and modified poly(A) RNAs 15nt in length were determined by Electrophoretic Mobility Shift Assay. PABPC at concentrations of 2000, 1000, 500, 250, 125, 63, 31, 16, 8, and 0 nM was incubated with unmodified poly(A) RNA at a concentration of 10 nM. PABPC at concentrations of 1000, 500, 250, 125, 63, 31, 16, 8, 4, and 0 nM were incubated with modified poly(A) RNA at a concentration of 5 nM. **(B)** Binding curves of PABPC to modified and unmodified poly(A) RNAs. The fraction of PABPC-bound RNA was plotted against the concentration of PABPC present. Binding curves and estimated Dissociation Constants ($K_D$) mean with standard deviation from three independent replicates are plotted in the graph.

## Supplemental Tables:

### Table S1: List and sequence of the different versions of Boosters

| oJC# | Booster ID | version | Alias | Sequence |
|---|---|---|---|---|
| 6480 | | 1 | Human LSM8-Booster1 | TTrUrGrUrArCrArArArGrArUrUrUrArCrArGrArUrGrUrCrCrArArGrUrA |
| 6285 | CB3 | 1 | CTNNB1-G1 | TGrUrCrArGrGrCrArCrUrUrUrCrUrGrArGrArUrArCrCrArGrCrCrCrArC |
| 6286 | CB4 | 1 | CTNNB1-G2 | CTrUrCrArCrUrUrCrUrUrGrArGrUrCrArCrUrCrCrCrArArArArUrCrCrA |
| 6287 | CB5 | 1 | CTNNB1-G3 | ATrUrArGrArArArUrUrGrCrUrGrUrArGrCrArGrUrArUrUrCrArCrUrArU |
| 6288 | CB6 | 1 | CTNNB1-G4 | TGrArUrCrArArArArCrArUrGrArArArUrArGrArUrCrCrArCrCrUrGrC |
| 6289 | CB7 | 1 | CTNNB1-G5 | CTrCrUrUrGrArArGrCrArUrCrGrUrArUrCrArCrArGrCrArGrGrUrUrArC |
| 7039 | SB1 | 2 | SynGAP-Booster1.1 | AAAAAAAAAAAAAAAAAAAAAAAAAAAAAAAAAAAAAAAAAAAAAAAAAA<br>CACCCTGAAGTTGAAAGTTTGGAGGTGCC |
| 7040 | SB2 | 2 | SynGAP-Booster1.3 | AAAAAAAAAAAAAAAAAAAAAAAAAAAAAAAAAAAAAAAAAAAAAAAAAA<br>CACCCTGAAGTTGAAAGTTTGGAGGTGCCAAAAAAAAAAAAAAAAAAAA<br>AAAAAAAAAAAAAAAAAAAAAAAAAAAAAAA |
| 7041 | SB3 | 2 | SynGAP-Booster2 | AAAAAAAAAAAAAAAAAAAAAAAAAAAAAAAAAAAAAAAAAAAAAAAAAA<br>ACCTCAATTCCACCCCACCCAACAAAAAAAAAAAAAAAAAAAAAAAAAA<br>AAAAAAAAAAAAAAAAAAAAAAAAA |
| 7042 | SB4 | 2 | SynGAP-Booster3 | AAAAAAAAAAAAAAAAAAAAAAAAAAAAAAAAAAAAAAAAAAAAAAAAAA<br>GAAACACCGAAAAATCGTGGAGCTGAAAAAAAAAAAAAAAAAAAAAAAA<br>AAAAAAAAAAAAAAAAAAAAAAAAAAA |
| 7043 | SB5 | 2 | SynGAP-Booster4 | AAAAAAAAAAAAAAAAAAAAAAAAAAAAAAAAAAAAAAAAAAAAAAAAAA<br>ATAAGTATAAATTTGGCCCCGGCAAAAAAAAAAAAAAAAAAAAAAAAAA<br>AAAAAAAAAAAAAAAAAAAAAAAAA |
| 7044 | SB6 | 2 | SynGAP-Booster5 | AAAAAAAAAAAAAAAAAAAAAAAAAAAAAAAAAAAAAAAAAAAAAAAAAA<br>AACCCCAGATAATTACAACAGCCAAAAAAAAAAAAAAAAAAAAAAAAAA<br>AAAAAAAAAAAAAAAAAAAAAAAAA |
| 6351 | control | 1 | random guide 3 | TTrArUrCrCrUrGrCrUrArCrGrGrArArGrCrUrCrArArUrCrArUrGrCrArU |
| 7034 | MB1 | 2 | MeCp2-Mouse2/Human | AAAAAAAAAAAAAAAAAAAAAAAAAAAAAAAAAAAAAAAAAAAAAAAAAA<br>GAGCCCACTTTAAAACAAGCGCAGGT |
| 7033 | MB2 | 2 | MeCp2-Mouse1/Human | AAAAAAAAAAAAAAAAAAAAAAAAAAAAAAAAAAAAAAAAAAAAAAAAAA<br>GAGCCCACTTTAAAACAAGCGCAGGTAAAAAAAAAAAAAAAAAAAAAAA<br>AAAAAAAAAAAAAAAAAAAAAAAAAA |
| 7035 | CB1 | 2 | CTNNB1-B1 | AAAAAAAAAAAAAAAAAAAAAAAAAAAAAAAAAAAAAAAAAAAAAAAAAA<br>CACTTTCTGAGATACCAGCCCACAAAAAAAAAAAAAAAAAAAAAAAAAA<br>AAAAAAAAAAAAAAAAAAAAAAAA |
| 7036 | CB2 | 2 | CTNNB1-B2 | AAAAAAAAAAAAAAAAAAAAAAAAAAAAAAAAAAAAAAAAAAAAAAAAAA<br>ACTTTGGGATAAAAGGCAACTGGAAAAAAAAAAAAAAAAAAAAAAAAAA<br>AAAAAAAAAAAAAAAAAAAAAAAA |

| | | | | |
|---|---|---|---|---|
| 7037 | PB1 | 2 | Pur-A-B1 | AAAAAAAAAAAAAAAAAAAAAAAAAAAAAAAAAAAAAAAAAAAAAAAAAA TTCCACAATTGAATTGGCCACAAACAAAAAAAAAAAAAAAAAAAAAAAAA AAAAAAAAAAAAAAAAAAAAAAAAA |
| 7038 | PB2 | 2 | Pur-A-B2 | AAAAAAAAAAAAAAAAAAAAAAAAAAAAAAAAAAAAAAAAAAAAAAAAAA TAAACCAGATCTGAAAAGAGGCCTAAAAAAAAAAAAAAAAAAAAAAAAA AAAAAAAAAAAAAAAAAAAAAAAAA |
| 6539 | SB7 | 3 | SynGAP-Booster3 | mA*mA*mA*AAAAAAAAAAAAAAAAAAAAAAAAAAAAAAAAAAAAAAAAAA AAAAAAAAAAAAAAAAAAAAAAAAAAAAAAAAAAAAAAAAAAAAAAAA AAAAAGGACUGUGUGUCAGAGUAGGACAGAUC*mA*mA*mC |
| 6540 | CB7 | 3 | CTNNB1 - Booster5 | mA*mA*mA*AAAAAAAAAAAAAAAAAAAAAAAAAAAAAAAAAAAAAAAAAA AAAAAAAAAAAAAAAAAAAAAAAAAAAAAAAAAAAAAAAAAAAAAAAA AAAAACUCUUGAAGCAUCGUAUCACAGCAGGU*mU*mA*mC |
| 6541 | control | 3 | scramble RNA | mA*mA*mA*AAAAAAAAAAAAAAAAAAAAAAAAAAAAAAAAAAAAAAAAAA AAAAAAAAAAAAAAAAAAAAAAAAAAAAAAAAAAAAAAAAAAAAAAAA AAAAAUUAUCCUGCUACGGAAGCUCAAUCAUG*mC*mA*mU |
| 7045 | SB1 -U | 2 | SynGAP 1-control-polu | TTTTTTTTTTTTTTTTTTTTTTTTTTTTTTTTTTTTTTTTTTTTTTTTTTCACC CTGAAGTTGAAAGTTTGGAGGTGCCTTTTTTTTTTTTTTTTTTTTTTTTTT TTTTTTTTTTTTTTTTTTTTTTTT |
| 6483 | | 3 | pA15-Unmodified | 5’- AAAAAAAAAAAAAAA-Cy5 -3’ |
| 6705 | | 3 | 2'oMe_PSall_poly(A)15 | mA*mA*mA*mA*mA*mA*mA*mA*mA*mA*mA*mA*mA*mA*mA/Cy5/ |
| 6706 | | 3 | 2'oMe_PSthree_poly(A)15 | mA*mA*mA*AAAAAAAAAAAA/Cy5/ |
| 6724 | | 3 | SynGAP Booster mod 75A | mA*mA*mA*AAAAAAAAAAAAAAAAAAAAAAAAAAAAAAAAAAAAAAAAAA AAAAAAAAAAAAAAAAAAAAAAAAAAAAAAGGACUGUGUGUCAGAGUA GGACAGAUC*mA*mA*mC |
| 6725 | | 3 | SynGAP Booster mod 50A | mA*mA*mA*AAAAAAAAAAAAAAAAAAAAAAAAAAAAAAAAAAAAAAAAAA AAAAAAAAAAAAAAAAAAAAAAAAAAAAAAGGACUGUGUGUCAGAGUA GGACAGAUC*mA*mA*mC |
| 6726 | | 3 | SynGAP Booster mod 25A | mA*mA*mA*AAAAAAAAAAAAAAAAAAAAAAGGACUGUGUGUCAGAGUA GGACAGAUC*mA*mA*mC |
| 6727 | | 3 | SynGAP Booster mod 3A | mA*mA*mA*GGACUGUGUGUCAGAGUAGGACAGAUC*mA*mA*mC |
| oJC6352, oWH6361 | | | Fluc booster+PS+10A | TGCTCGAAGCGGCCGGCCGCCCCGA/iSp9/rArArArArArArArArArA |
| oJC6353 oWH6362 | | | Fluc booster, no A, control | TGCTCGAAGCGGCCGGCCGCCCCGA |

**Table S2: List and the sequence of oligos used for PCR and qPCR analysis**

| oJC# | Alias | Sequence |
|---|---|---|
| 6529 | LSM8-forward | CGAGTATTCAGCTCTTCACAGGG |
| 6530 | LSM8-Reverse | CCCAAATCAAGCGCAGAATCTGT |
| 6531 | EN2-forward | GCTTGTCCTCTTTGTTCGGGTTC |
| 6532 | EN2-Reverse | CAACCTGGGCGCGCAGCCCAT |
| 6544 | DKK1-F | GGTATTCCAGAAGAACCACCTTG |
| 6545 | DKK1-R | CTTGGACCAGAAGTGTCTAGCAC |
| 6546 | C-myc-F | CCTGGTGCTCCATGAGGAGAC |
| 6547 | C-myc-R | CAGACTCTGACCTTTTGCCAGG |
| 6638 | Syngap1-F | CAACCTGCGAATGTGCTGTGAG |
| 6639 | Syngap1-R | CGCTGATAAGCCTGTCTGCGAT |
| 6911 | Syngap1 F (mouse) | CCGGACCAGCAGCTTTC |
| 6912 | Syngap1 R(mouse) | CCCAGGATGGAGCTGTG |
| 6913 | Actin F (mouse) | TCCTTCTGACCCATTCCCA |
| 6914 | Actin R (mouse) | TCCGGCATGTGCAAAGC |
| 5544 | Mecp2-F | TGAAGGCTGGACACGGAAGCTT |
| 5545 | Mecp2-R | CAGGGATGTGTCGCCTACCTTT |
| 7046 | PurA-F | CTCCTTGACTGTGGACAACAAG |
| 7047 | Pur-A-R | GCAGAAGGTGTGTCCGAACTTG |
| 7048 | CTNNB1-F | CAAGCAGAGTGCTGAAGGTGCTA |
| 7049 | CTNNB1-R | GATTCCTGAGAGTCCAAAGACAG |
| 5067 | Actin-F | CACCATTGGCAATGAGCGGTTC |
| 5068 | Actin-R | AGGTCTTTGCGGATGTCCACGT |
| 6380 | GAPDH-F | AATCCCATCACCATCTTCCA |
| 6381 | GAPDH-R | TGGACTCCACGACGTACTCA |
| 7050 | M13-F | TGTAAAACGACGGCCAGT |
| 7051 | M13-R | CACAGGAAACAGCTATGA |
| 6730 | SYN3 PROB | GTTGATCTGTCCTACTCTGACACACAGTC |
| 7078 | M.Mecp2F1 | AGGAAGTCTGGCCGATCTG |
| 7079 | M.MeCp2R1 | TCATTAGGGTCCAAGGAGGT |
| 7080 | M.Mecp2F2 | GCTGGGATGTTAGGGCTCA |
| 7081 | M.Mecp2R2 | CTGAAGGTTGTAGTGGCTCAT |
| 7082 | M-nfkb1-F | GCTGCCAAAGAAGGACACGACA |
| 7083 | M-nfkb1-R | GGCAGGCTATTGCTCATCACAG |
| 7084 | M-TNFa-F | GGTGCCTATGTCTCAGCCTCTT |
| 7085 | M-TNFa-R | GCCATAGAACTGATGAGAGGGAG |

| | | |
|---|---|---|
| oJC6339, oWH6357 | amplify off pWH231 | GCTAATACGACTCACTATAGGTACTGTTGGTAAAG CCACCATGGAAG |
| oJC6340, oWH6358 | amplify off pWH231 | GGTTTGTCCAAACTCATCAATGTATCTTATCATGTC |
| oJC6341, oWH6359 | amplify off pWH232 | GCTAATACGACTCACTATAGGCTAGCCACC |
| oJC6342, oWH6360 | amplify off pWH232 | TTTTATTGCGGCCGCTTATTGTTCATTTTTGAGAAC TCGCTCAACGAA |

**Table S3: List of the plasmids**

| pJC# | Plasmid name | Description |
|---|---|---|
| 315 | pbluescript | backbone |
| 1294 | Mecp2 1tail-polyA | MB1 |
| 1295 | Mecp2 2tail-polyA | MB2 |
| 1419 | mouse mecp2-g1-2tail | MB3 |
| 1420 | mouse mecp2-g2-2tail | MB4 |
| 1417 | syngap1-1tail guide | SB1 |
| 1418 | syngap1-2tail guide | SB2 |
| 1459 | SynGAP1-Booster1-polU | SBU |
| 1460 | SynGAP1-PolA-booster2-PolA | SB3 |
| 1461 | SynGAP1-PolA-booster3-PolA | SB4 |
| 1462 | SynGAP1-PolA-booster4-PolA | SB5 |
| 1463 | SynGAP1-PolA-booster5-PolA | SB6 |
| 1464 | PurA-polA-boostert1-PolA | PB1 |
| 1465 | PuaA-polA-booster2-polA | PB2 |
| 1466 | CTNNB1-polA-booster1-polA | CB1 |
| 1467 | CTNNB1-polA-booster2-polA | CB2 |
| pJC1658, pWH231 | pRL-SV40 (T7 promoter RLuc vector) | |
| pJC1659, pWH232 | pGL4.10[luc2] (Fluc vector) | |

**Table S4: Formulation details and particle sizes for the LNPs**

| Code | Mol % | | | | N/P Ratio | Z-Average (nm) | PDI |
|---|---|---|---|---|---|---|---|
| | Helper lipid | Dlin-MC3 | Chol | DMG-PEG | | | |
| **C9 (brain)** | 40 | 40 | 19.80 | 0.02 | 8 | 124.31 ± 2.93 | 0.21 ± 0.04 |
| **FIII-7 (liver)** | 5.45 | 54.55 | 39.92 | 0.08 | 4 | 132.23 ± 3.76 | 0.14 ± 0.03 |
